# The EXERRT trial: “EXErcise to Regadenoson in Recovery Trial”: A phase 3b, open-label, parallel group, randomized, multicenter study to assess regadenoson administration following an inadequate exercise stress test as compared to regadenoson without exercise for myocardial perfusion imaging using a SPECT protocol

**DOI:** 10.1007/s12350-017-0813-3

**Published:** 2017-02-21

**Authors:** Gregory S. Thomas, S. James Cullom, Therese M. Kitt, Kathleen M. Feaheny, Karthikeyan Ananthasubramaniam, Robert J. Gropler, Diwakar Jain, Randall C. Thompson

**Affiliations:** 1 MemorialCare Heart & Vascular Institute, Long Beach Memorial, 2801 Atlantic Ave, Long Beach, CA 90806 USA; 2 University of California, Irvine, CA USA; 3 AdaptivePharma, Leawood, KS USA; 4 University of Missouri, Columbia, MO USA; 5 Astellas Pharma Global Development, Inc., Northbrook, IL USA; 6 Department of Internal Medicine, Heart and Vascular Institute, Henry Ford Hospital, Detroit, MI USA; 7 Division of Radiological Sciences, Mallinckrodt Institute of Radiology, Washington University School of Medicine, St Louis, MO USA; 8 Cardiovascular Nuclear Imaging Laboratory, New York Medical College, Westchester Medical Center, Valhalla, NY USA; 9 Saint Luke’s Mid America Heart Institute, Kansas City, MO USA; 10 University of Missouri-Kansas City, Kansas City, MO USA

**Keywords:** Exercise, pharmacologic stress, vasodilator stress, myocardial perfusion imaging, regadenoson

## Abstract

**Background:**

This study assessed the non-inferiority and safety of regadenoson administration during recovery from inadequate exercise compared with administration without exercise.

**Methods:**

Patients unable to achieve adequate exercise stress were randomized to regadenoson 0.4 mg either during recovery (Ex-Reg) or 1 hour after inadequate exercise (Regadenoson) (MPI1). All patients also underwent non-exercise regadenoson MPI 1-14 days later (MPI2). The number of segments with reversible perfusion defects (RPDs) detected using single photon emission computerized tomography imaging was categorized. The primary analysis evaluated the majority agreement rate between Ex-Reg and Regadenoson groups.

**Results:**

1,147 patients were randomized. The lower bound of the 95% confidence interval of the difference in agreement rates (−6%) was above the −7.5% non-inferiority margin, demonstrating non-inferiority of Ex-Reg to Regadenoson. Adverse events were numerically less with Ex-Reg (MPI1). In the Ex-Reg group, one patient developed an acute coronary syndrome and another had a myocardial infarction following regadenoson after exercise. Upon review, both had electrocardiographic changes consistent with ischemia prior to regadenoson.

**Conclusions:**

Administering regadenoson during recovery from inadequate exercise results in comparable categorization of segments with RPDs and with careful monitoring appears to be well tolerated in patients without signs/symptoms of ischemia during exercise and recovery.

**Electronic Supplementary Material:**

The online version of this article (doi:10.1007/s12350-017-0813-3) contains supplementary material, which is available to authorized users.

## Introduction

Exercise or pharmacological stress myocardial perfusion imaging (MPI) is an integral part of the non-invasive evaluation of patients with suspected or known coronary artery disease (CAD). Patients who are ambulatory and able to walk on a treadmill are often referred for exercise MPI. However, the diagnostic accuracy of exercise MPI studies is suboptimal in patients unable to achieve 85% of maximum predicted heart rate (MPHR) and 5 metabolic equivalents (METs).^1^ Ambulatory patients who are not expected to achieve adequate stress are often referred for pharmacologic stress testing combined with low-level or symptom-limited exercise.^2^-^14^ This approach has been shown to be well tolerated, improve image quality, and diminish side effects.^5^,^11^-^14^ Moreover, the addition of symptom-limited exercise has also been shown to generate incremental prognostic data complementary to MPI results.^2^ However, it is sometimes difficult to predict whether a patient will achieve adequate exercise stress. When exercise is inadequate, changing to pharmacological stress with agents such as adenosine or dipyridamole can involve delays associated with preparation for infusion. In turn, that can disrupt lab scheduling and potentially require rescheduling of the test to another day.^1^ The availability of the pharmacologic stress agent regadenoson, which is administered as a fixed-dose rapid injection, creates the opportunity for its use as an adjunctive stress agent in patients who undergo exercise testing and fail to achieve adequate exercise stress.^7^-^9^,^13^,^15^-^18^ Single center studies using regadenoson in combination with exercise have been generally favorable;^3^,^6^-^9^,^12^,^13^ however, adverse reactions have been reported.^13^,^15^ Nevertheless, the comparability of this approach with the administration of regadenoson without exercise has not previously been investigated in a large clinical trial.

In order to investigate the assessment of reversible perfusion defects (RPDs) and the safety when regadenoson is administered during recovery following inadequate exercise stress, we conducted the multicenter, multicountry, open-label, randomized parallel design clinical trial described herein. The objectives of this study were to demonstrate that the strength of agreement between single photon emission computed tomography (SPECT) imaging with regadenoson administered during recovery following inadequate exercise stress testing and regadenoson SPECT imaging without exercise was not inferior to the strength of agreement between two sequential regadenoson SPECT images without exercise and to assess safety.

## Methods

### Participants

The EXERRT study was conducted between June 29, 2012 and December 14, 2014 in the United States (44 centers), Argentina (4 centers), and Peru (1 center). In order to be enrolled in this phase 3b study (ClinicalTrials.gov identifier, NCT01618669), patients must have been referred for a clinically indicated exercise or pharmacologic stress SPECT MPI for the evaluation of CAD. Based on the opinion of the investigator, patients were to have a reasonable potential of attempting exercise stress. Patients were excluded if they had high-risk unstable angina, acute myocardial infarction (MI) within 30 days, coronary revascularization within 1 month, a history of second- or third-degree atrioventricular (AV) block or left bundle branch block, pacemaker, or implantable cardioverter defibrillator. MET levels were estimated and not measured directly. Caffeine-containing foods and beverages were withheld for 12 hours prior to the administration of regadenoson. Each site followed their local protocol regarding beta blockers. Full inclusion and exclusion criteria are listed in the Electronic Supplementary Material.

The study was conducted in compliance with the principles of the Declaration of Helsinki, International Conference on Harmonization of Technical Requirements for Registration of Pharmaceuticals for Human Use, and Good Clinical Practice. The institutional review board or independent ethics committee of each study center approved the protocol and consent form. Each participant provided written informed consent.

### Study Design

#### Stress Testing and Imaging

**Figure 1:**
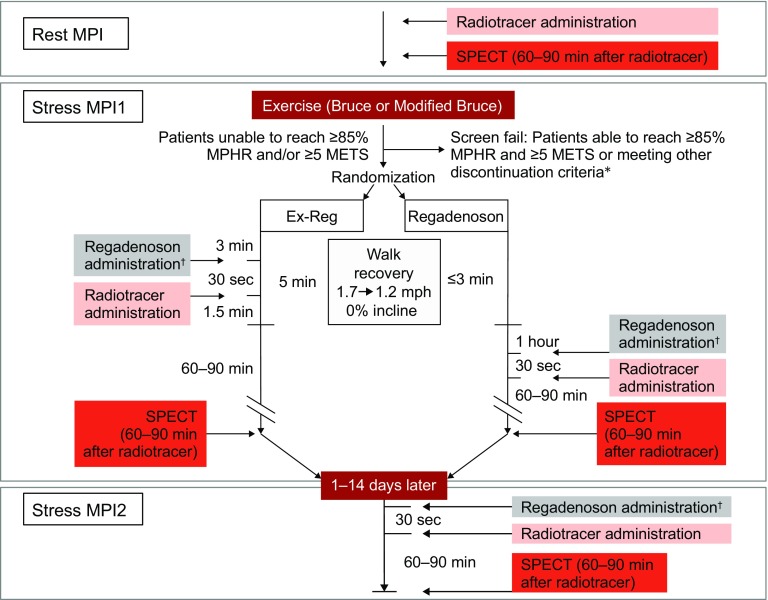
Illustration of the flow of patients through all stages of the study. *As described in the Methods section, patients experiencing signs or symptoms of ischemia prior to receiving regadenoson were not to be randomized. ^†^Administered intravenously over 10 seconds. *MPI*, Myocardial perfusion imaging; *SPECT*, single photon emission computed tomography

Following a baseline visit, patients underwent resting SPECT MPI in accordance with American Society of Nuclear Cardiology 2009 guidelines (Fig. 1).^1^ For 1-day studies, sites were instructed to use 4-12 mCi for the rest scan and 13-36 mCi for the stress scan; for 2-day studies, sites were instructed to use 13-36 mCi for the rest scan and 13-36 mCi for the stress scan. Patients then initiated exercise using a standard or modified Bruce protocol.^19^,^20^ If the patient achieved ≥85% of MPHR and ≥5 METs of activity, the patient was discontinued from the study. If the patient did not achieve ≥85% of MPHR or ≥5 METs of activity or both, and did not meet other discontinuation criteria, they transitioned into a 3- to 5-minute walking recovery. During the first 3 minutes of recovery, patients were randomized 1:1 to either regadenoson following exercise (Ex-Reg group) or regadenoson (Regadenoson group). As shown in Fig. 1, Ex-Reg patients received regadenoson at 3 minutes of the walking recovery while Regadenoson patients received regadenoson at rest 1 hour later (to allow hemodynamics to return to baseline). SPECT imaging was performed 60-90 minutes after regadenoson administration in each group. All patients returned 1-14 days later to undergo a second regadenoson stress study without exercise. The first regadenoson scan and the baseline resting scan comprised MPI1 and the second regadenoson scan 1-14 days later and the same baseline resting scan comprised MPI2. Details of image processing and analysis are included in the Electronic Supplementary Material.

Images were processed at an independent core laboratory (ICON, Doylestown, PA), where they were interpreted by three expert nuclear cardiology readers who were blinded to randomization protocol, test performance, and medical history. Images were scored using a 17-segment model with a 5-point perfusion scale ranging from 0 = normal perfusion to 4 = absent uptake.^21^ Segments were counted as having a reversible defect if the stress score was greater than the rest score and the stress score was ≥2 (2 = moderately reduced radiotracer uptake). Two or more segments were required to meet the criteria for ischemia. This definition of reversible defect was prospectively defined in the protocol and statistical plan before any patient images were viewed by the blinded readers. Planar imaging in the anterior view was performed immediately following each stress SPECT scan to assess the target-to-background ratio of heart-to-liver, heart-to-gut, and heart-to-(the mean of) liver and gut.^12^,^22^ Data on radiation exposure were collected.

Patients underwent 12-lead Holter monitoring during testing, analyzed by a core laboratory for arrhythmia and 12-lead electrocardiogram (ECG) interpretation (ERT, Philadelphia, PA). Patients were also monitored during the imaging procedures for heart rate, blood pressure, and adverse events.

#### Criteria for Trial Discontinuation

Criteria for discontinuation from the trial during exercise testing were modified during the study. Initially, if a patient met an absolute or relative indication to terminate exercise testing based on the American College of Cardiology/American Heart Association 2002 guidelines, the investigator was to make a clinical judgment whether to continue the patient in the trial (i.e., proceed with randomization).^20^ Following a report of an acute coronary syndrome (ACS), the protocol was amended to require discontinuation in patients experiencing signs or symptoms of ischemia during exercise or recovery prior to regadenoson administration. Full criteria for termination from the study are listed in the Electronic Supplementary Material.

#### Endpoints

The primary endpoint used the number of segments with RPDs (referred to as ischemia for this trial) categorized as absent (0-1 segment) or present (≥2 segments) as assessed by each of the three readers. Each reader was defined as having self-agreement based upon identical categorization of a given patient as follows: 0-1 segment with RPDs (absence of ischemia) for both MPI1 and MPI2; ≥2 segments with RPDs (presence of ischemia) for both MPI1 and MPI2. A given patient was then defined as having a majority agreement of Yes if at least 2 of the 3 readers demonstrated self-agreement. The primary endpoint was the binary outcome of majority agreement of Yes or No for reader self-agreement.

The safety composite variable was defined as the percentage of patients who experienced at least one treatment-emergent clinically significant cardiac event. The safety composite, secondary and safety endpoints, and the planned analyses are described in the Electronic Supplementary Material.

### Statistical Methodology

A sample size of 450 evaluable patients in each group completing MPI1 and MPI2 would provide 90% power using an alpha level of 5% to demonstrate non-inferiority at a margin of 7.5%. In determining the non-inferiority margin, each group was assumed to have a majority agreement rate of 86% based on the regadenoson pivotal studies data.^17^ Assuming a 20% dropout rate, approximately 1,130 patients would need to be randomized.

The efficacy analysis set included all randomized patients with interpretable MPI1 and MPI2 scans as determined by at least two of three readers. The safety analysis set included all randomized patients who received at least one dose of regadenoson.

The agreement rate for each group was calculated as the number of patients where the majority of readers agreed on their individual assessment of the two stress MPI scans divided by the total number of patients in the group. The primary assessment of the non-inferiority hypothesis was provided by a confidence interval (CI) on the difference in agreement rates (Ex-Reg agreement rate minus Regadenoson agreement rate). The CI was calculated using the Newcombe score methodology.^23^ The lower confidence bound of the one-sided alpha level of 0.025 of the difference in agreement rates was to exceed −7.5% in order to demonstrate non-inferiority. The primary efficacy assessment was performed for the efficacy analysis set. Statistical methods for secondary endpoints are provided in the Electronic Supplementary Material.

### Role of the Funder

The funder of the study, Astellas Pharma Global Development, Inc., was involved in study design, data collection, data analysis, data interpretation, and writing of the report. The corresponding author had full access to all data in the study and all authors had final responsibility for the decision to submit for publication.

## Results

### Patients

**Figure 2:**
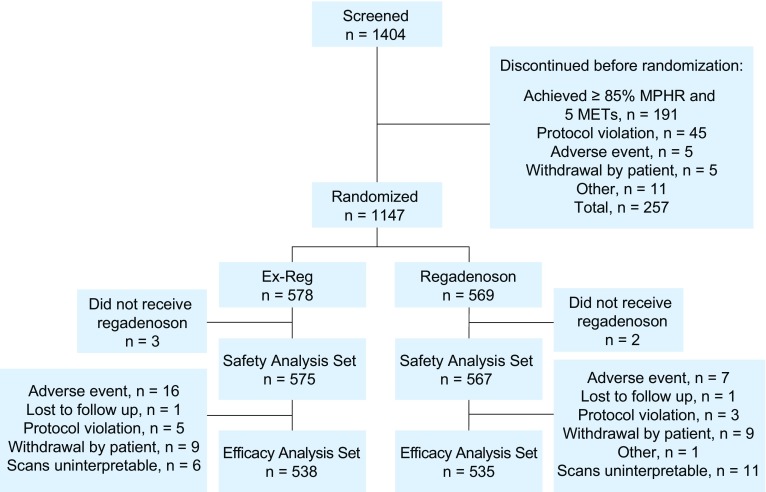
Diagram outlining the flow of patients in the study, including events that precluded patients from analysis. Reasons for exclusion from efficacy analysis set were factors that prevented completion of all MPI assessments. *MET*, Metabolic equivalent; *MPHR*, maximum predicted heart rate

Of the 1,147 patients randomized, the efficacy analysis set included 538 patients in the Ex-Reg group and 535 patients in the Regadenoson group (Fig. 2). Demographics, cardiac history, and test referral were comparable between the Ex-Reg and Regadenoson groups (Table 1). The Bruce protocol was used in 83% and the modified Bruce protocol in the remainder. Patients achieved 5.4 ± 2.3 METs and a mean heart rate that was 63.6% ± 4.6% of the MPHR. Rest and stress testing was performed on the same day in 96% of patients.

**Table 1:** Baseline characteristics (safety analysis set)

Parameter	Ex-Reg (*n* = 575)	Regadenoson (*n* = 567)
Age (years)	62 ± 11	62 ± 11
Male	341 (59.3)	328 (57.8)
Race		
White	456 (79.3)	441 (77.8)
Black or African American	71 (12.3)	83 (14.6)
Asian	42 (7.3)	37 (6.5)
Other	6 (1.0)	6 (1.1)
BMI (kg/m^2^)	31 ± 7	31 ± 7
Cardiovascular history		
Hypertension	504 (87.7)	489 (86.2)
Dyslipidemia	434 (75.5)	414 (73.0)
CAD	333 (57.9)	303 (53.4)
Diabetes	198 (34.4)	194 (34.2)
Previous PCI	187 (32.5)	171 (30.2)
Previous MI	145 (25.2)	122 (21.5)
Current smoker	139 (24.2)	125 (22.0)
Previous CABG	80 (13.9)	74 (13.1)
Peripheral vascular disease	64 (11.1)	53 (9.3)
Congestive heart failure	28 (4.9)	23 (4.1)
Referred for pharmacologic stress test only	274 (47.7)	257 (45.3)
Referred for exercise stress test only	270 (47.0)	270 (47.6)
Referred for pharmacologic and exercise stress tests	31 (5.4)	40 (7.1)
Exercise protocol*		
Bruce protocol	448 (83.3)	442 (82.6)
Duration of exercise (min)	4.1 ± 2.3	4.1 ± 2.2
Percent of MPHR	63.2 ± 4.6	63.4 ± 4.5
Maximum METs achieved	5.9 ± 2.2	5.9 ± 2.2
Modified Bruce protocol	90 (16.7)	93 (17.4)
Duration of exercise (min)	4.7 ± 2.6	4.3 ± 2.6
Percent of MPHR	65.5 ± 4.4	64.7 ± 4.3
Maximum METs achieved	3.1 ± 1.1	2.9 ± 1.3
All patients (Combined Bruce or Modified Bruce)	538	535
Percent of MPHR	63.6 ± 4.6	63.6 ± 4.5
Maximum METs achieved	5.5 ± 2.3	5.4 ± 2.3

All values are mean ± SD or *n* (%)

*BMI*, Body mass index; *CABG*, coronary artery bypass grafting; *CAD*, coronary artery disease; *MET*, metabolic equivalent; *MI*, myocardial infarction; *MPHR*, maximum predicted heart rate; *PCI*, percutaneous coronary intervention; *SD*, standard deviation; *SPECT*, single photon emission computed tomography

*Percentages based on the efficacy analysis set (Ex-Reg, *N* = 538; Regadenoson, *N* = 535). Safety analysis set = patients who received at least one dose of regadenoson during the study. Efficacy analysis set = all randomized patients who received regadenoson study drug with interpretable SPECT scans at all visits as determined by at least two of the three blinded expert readers

### Primary Efficacy Endpoint

**Figure 3:**
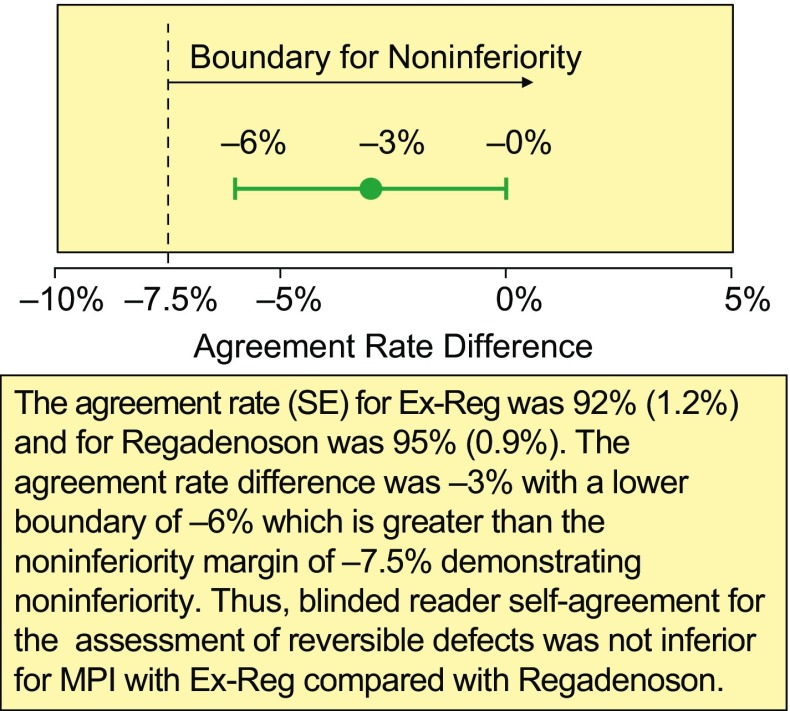
Primary endpoint: Majority agreement rate difference (results of the primary endpoint). *MPI*, Myocardial perfusion imaging; *SE*, standard error

In the primary analysis, majority agreement rates (95% CI) for the Ex-Reg and Regadenoson groups were 92% (89%, 94%) and 95% (93%, 97%), respectively. The difference in the majority agreement rates (Ex-Reg—Regadenoson) was −3% (95% CI: −6%, −0%). The lower bound (−6%) was above the non-inferiority margin of −7.5%, demonstrating that the agreement rate for the Ex-Reg group was not inferior to the agreement rate for the Regadenoson group (Table 2; Fig. 3). Thus, for reader self-agreement of assessment of RPDs, Ex-Reg (regadenoson administered 3 minutes post exercise during recovery at MPI1) was not inferior to Regadenoson (regadenoson administered at rest for MPI1).

**Table 2:** Majority agreement between MPI1 and MPI2

Values are based on efficacy analysis set

*CI*, Confidence interval; *MPI*, myocardial perfusion imaging

*If the lower confidence bound of the one-sided alpha level of 0.025 of the difference in agreement rates exceeded −7.5%, non-inferiority was demonstrated

### Secondary Efficacy Endpoints

The agreement rate analysis using the median assessment of number of segments with RPDs across the three readers categorized using two categories (0-1, ≥2) did not demonstrate non-inferiority. Using three categories (0-1, 2-4, ≥5), non-inferiority could not be assessed because of insufficient data (i.e., there were no Regadenoson group patients with ≥5 RPDs at MPI1) (Table 3). Based on the median assessment, ≥91% of patients had 0-1 segment with RPDs. Reader interpretation was similar between the Ex-Reg and Regadenoson groups in the evaluation of Summed Stress Scores (SSS) categorized as 0-3, 4-7, 8-11, and ≥12 and Summed Difference Scores (SDS) using the pre-defined broad categories of 0-6, 7-13, and ≥14 (Electronic Supplementary Material Table 1) and categorized post hoc as 0-2, 3-6, 7-13, and ≥14 as shown in Table 4.

**Table 3:** Agreement rates for MPI

Blue highlighting added to improve clarity of overall table organization and to accentuate categories with agreement between MPI1 and MPI2

*CI*, Confidence interval; *MPI*, myocardial perfusion imaging; *NC*, not calculated

*Agreement rate differences were calculated as the Ex-Reg agreement rate minus the Regadenoson agreement rate

^†^The calculation of the “All” agreement rate for Ex-Reg and Regadenoson was based on the 0-1 and 2-4 categories. The ≥5 category was not included because of a lack of data available for the Regadenoson ≥5 category for MPI1. In addition, the insufficient data for Regadenoson did not permit an agreement rate to be calculated for this row, as indicated by “NC”

**Table 4:** Summed stress scores agreement rates and summed difference scores

Blue highlighting added to improve clarity of overall table organization and to accentuate categories with agreement between MPI1 and MPI2

*CI*, Confidence interval; *MPI*, myocardial perfusion imaging; *SDS*, Summed Difference Score; *SE*, standard error; *SSS*, Summed Stress Score

*Agreement rate differences were calculated as the Ex-Reg agreement rate minus the Regadenoson agreement rate

^†^Cohen’s Kappa and weighted Kappa statistics

Side-by-side reader assessment of the number of RPDs comparing MPI1 to MPI2 in the Ex-Reg group (*n* = 538) showed fewer RPDs in 10.6%, the same in 82.0%, and more in 7.4% of patients (*P* = 0.104). In the Regadenoson group (*n* = 535), fewer RPDs were observed in 9.2%, 85.8% were the same, and more in 5.0% of patients (*P* = 0.015).

Target-to-background ratios were higher for stress MPI1 in the Ex-Reg group compared to the arm when regadenoson was given at rest (Table 5). Stress MPI image quality was similar between the groups, assessed as excellent/good in ≥92% of patients in both groups with uninterpretable scans in 1.5% of patients (Electronic Supplementary Material Table 2). Subdiaphragmatic activity interfering with image quality was less common on stress MPI1 than stress MPI2 in Ex-Reg (*P* = 0.019) and not different between stress MPI1 and MPI2 in Regadenoson (*P* = 0.921) (Electronic Supplementary Material Table 3).

**Table 5:** Heart-to-background ratios compared within each group

Ratio	Stress	Stress		
	MPI1	MPI2	Difference (95% CI*)	*P* value^†^
Ex-Reg (n = 538)				
Heart-to-liver	1.05 (0.40)	0.94 (0.37)	0.10 (0.10 to 0.10)	<0.001
Heart-to-gut	1.12 (0.44)	0.99 (0.40)	0.15 (0.10 to 0.20)	<0.001
Heart-to-liver/gut	1.02 (0.30)	0.90 (0.26)	0.10 (0.10 to 0.10)	<0.001
Regadenoson (n = 535)				
Heart-to-liver	0.96 (0.37)	0.95 (0.36)	0.00 (0.00 to 0.00)	
Heart-to-gut	1.05 (0.43)	0.99 (0.39)	0.05 (0.00 to 0.10)	NC
Heart-to-liver/gut	0.94 (0.27)	0.91 (0.26)	0.05 (0.00 to 0.10)	

All values are mean ± SD

*CI*, Confidence interval; *MPI*, myocardial perfusion imaging; *NC*, not calculated; *SD*, standard deviation

*Hodges-Lehmann CI

^†^Wilcoxon signed-rank test for Ex-Reg only

### Safety

#### Adverse Events

Serious adverse events occurring within 24 hours of regadenoson administration were reported for five patients (0.9%) in the Ex-Reg group during MPI1, two patients (0.4%) in the Ex-Reg group during MPI2, and one patient (0.2%) in each of the MPIs for the Regadenoson group. All adverse events occurring at a frequency ≥5% are reported in Table 6. Fifty-three percent of the patients reported an adverse event when regadenoson was administered 3 minutes into recovery compared with 58% to 59% of patients when regadenoson was given without exercise. Headache and flushing were numerically less in the Ex-Reg group during MPI1. Clinically significant cardiovascular events occurred in three patients and are discussed below.

**Table 6:** Treatment-emergent adverse events (safety analysis set)

TEAEs, n (%)	Ex-Reg		Regadenoson	
	Stress MPI1 Regadenoson Following Exercise (*n* = 575)	Stress MPI2 Regadenoson (*n* = 544)	Stress MPI1 Regadenoson (*n* = 567)	Stress MPI2 Regadenoson (*n* = 548)
Any TEAE	302 (52.5)	317 (58.3)	329 (58.0)	323 (58.9)
Drug-related TEAEs*	291 (50.6)	298 (54.8)	319 (56.3)	308 (56.2)
TEAEs leading to discontinuation	13 (2.3)	0	5 (0.9)	1 (0.2)
Deaths	0	0	0	0
Serious TEAEs^†^	5 (0.9)	2 (0.4)	1 (0.2)	1 (0.2)
ACS	1 (0.2)	0	0	0
Congestive heart failure	0	0	1 (0.2)	0
MI	1 (0.2)	0	0	0
Myocardial ischemia	1 (0.2)	1 (0.2)	0	0
Vision blurred	1 (0.2)	0	0	0
Pancreatitis	0	1 (0.2)	0	0
Subtherapeutic INR	0	0	0	1 (0.2)
Abnormal hepatic enzymes	1 (0.2)	0	0	0
Dizziness	1 (0.2)	0	0	0
Speech disorder	1 (0.2)	0	0	0
Syncope	1 (0.2)	0	0	0
Most common TEAEs^‡^				
Dyspnea	141 (24.5)	125 (23.0)	161 (28.4)	152 (27.7)
Headache	85 (14.8)	108 (19.9)	137 (24.2)	118 (21.5)
Dizziness	107 (18.6)	75 (13.8)	89 (15.7)	81 (14.8)
Flushing	47 (8.2)	78 (14.3)	79 (13.9)	69 (12.6)
Nausea	43 (7.5)	44 (8.1)	45 (7.9)	41 (7.5)
Chest discomfort	37 (6.4)	33 (6.1)	54 (9.5)	43 (7.8)
Abdominal pain upper	31 (5.4)	35 (6.4)	35 (6.2)	34 (6.2)
Dysgeusia	16 (2.8)	27 (5.0)	25 (4.4)	23 (4.2)
Treatment-emergent clinically significant cardiac events				
Any cardiac event	17 (3.0)	5 (0.9)	3 (0.5)	2 (0.4)
Any ECG abnormality^§^	16 (2.8)	5 (0.9)	3 (0.5)	2 (0.4)
ST-T depression (≥2 mm)	13 (2.3)	3 (0.6)	2 (0.4)	2 (0.4)
ST-T elevation (≥1 mm)	3 (0.5)	2 (0.4)	1 (0.2)	0
Major cardiac adverse events	2 (0.3)	0	0	0
ACS	1 (0.2)	0	0	0
MI	1 (0.2)	0	0	0
Adverse event of unstable angina	0	0	0	0

*ACS,* acute coronary syndrome; *AV,* atrioventricular; *ECG,* electrocardiogram; *INR,* international normalized ratio; *MI,* myocardial infarction; *MPI,* myocardial perfusion imaging; *TEAE,* treatment-emergent adverse event occurring within 24 hours of regadenoson administration

*Considered as possibly or probably related to study drug by the study investigator. ^†^One patient experienced multiple serious TEAEs. ^‡^Events occurring in ≥5% of patients in any group. ^§^Other ECG abnormalities that were considered included sustained ventricular tachycardia, ventricular fibrillation or ventricular flutter, torsade de pointes, 2:1 AV block, Mobitz I second-degree AV block, Mobitz II second-degree AV block, complete heart block and pause >3.0 seconds; none of these were reported for any patient

A 55-year-old man (Ex-Reg MPI1) was referred for evaluation of jaw pain. He exercised for 5 minutes and achieved 7.1 METs and 65% MPHR on the Bruce protocol. During exercise he developed jaw pain and downsloping inferolateral ST segment depression. Following administration of regadenoson, he experienced chest pain and inferior ST elevation. Symptoms improved after nitroglycerin. He underwent urgent coronary angiography demonstrating two-vessel CAD with a subtotal right coronary artery (RCA) lesion with thrombus. RCA aspiration thrombectomy and stenting were successful; troponins were negative, and MI was excluded. Aminophylline was not administered in this patient. Criteria for discontinuation were amended following this ACS event.

A 65-year-old man (Ex-Reg MPI1) with history of MI, coronary artery bypass graft surgery, and diabetes mellitus exercised for 5 minutes on the Bruce protocol achieving 6.2 METs and 48% MPHR. During early recovery, lateral ST depression (<1 mm) and inferior ST elevation (>1 mm) developed. He received regadenoson, developing chest pain, dizziness, and dyspnea with increasing inferior ST elevation. Symptoms resolved and he was sent home. He presented 7.5 hours later with an ST elevation MI. The investigator read ischemia (4 RPDs) on SPECT imaging. Aminophylline was not administered in this patient.

A 56-year-old man experienced chest tightness and ST and T wave changes after stage 1 of the Bruce protocol. Exercise was stopped and his symptoms resolved. He was not randomized and did not receive regadenoson. Coronary angiography revealed a subtotal RCA occlusion that was treated with a coronary stent.

The safety composite variable, defined as the percentage of patients who experienced at least one treatment-emergent clinically significant cardiac event, is summarized in Table 6. Overall, ≤3% of patients in each group experienced a significant cardiac event. Thirteen patients in the Ex-Reg group and two patients in the Regadenoson group showed ST depression ≥2 mm during stress MPI1 and three patients in the Ex-Reg group and one patient in the Regadenoson group showed ST elevation ≥1 mm during stress MPI1. During stress MPI2, three patients in the Ex-Reg group and two patients in the Regadenoson group showed ST depression ≥2 mm and two patients in Ex-Reg showed ST elevation ≥1 mm. In general, the changes in ST segments were transient and did not result in a serious cardiac event with the exception of the 65-year-old patient discussed above. Of note, no cases of second- or third-degree heart block or asystole were observed in the 1,142 patients who received regadenoson in the trial on either the site ECG or the core laboratory Holter recording.

#### Heart Rate and Blood Pressure

**Figure 4:**
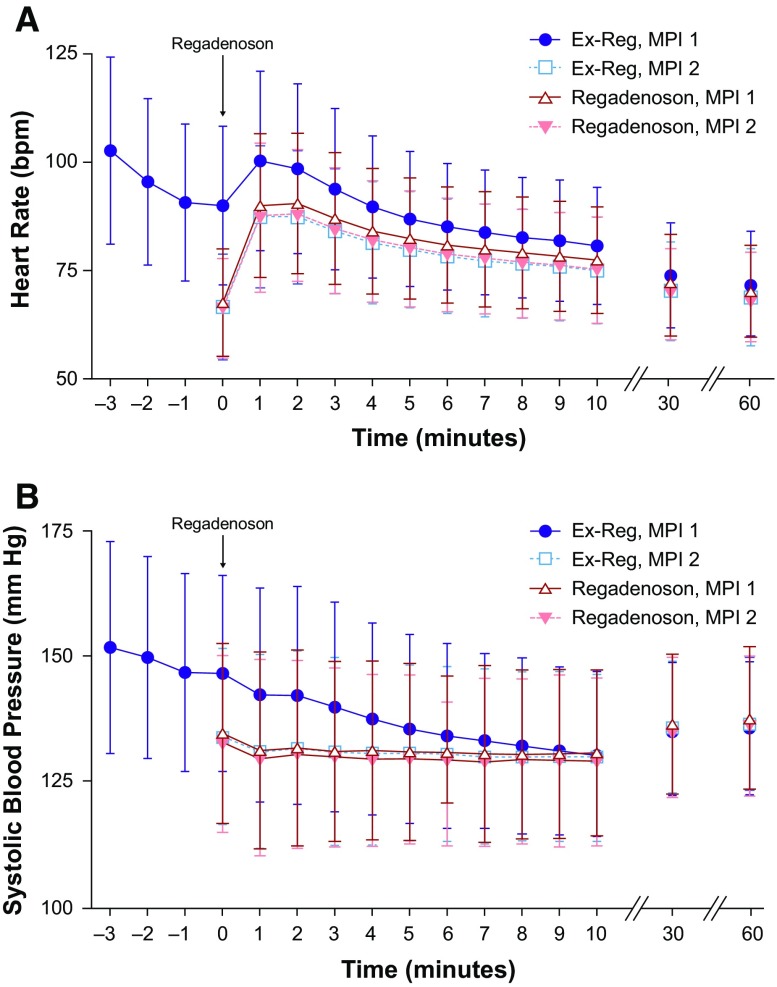
Responses for heart rate (**A**) and systolic blood pressure (**B**). Illustration of the mean and SD changes in heart rates (**A**) and systolic blood pressure (**B**) over 60 minutes in Ex-Reg and Regadenoson during the first and second stress MPI procedures. *MPI*, Myocardial perfusion imaging; *SD*, standard deviation

Heart rate increased by a mean of 10 ± 15 beats per minute (BPM) following regadenoson administration during walk recovery (Ex-Reg MPI1) and a mean of 21-22 BPM when administered at rest (Fig. 4A). A decrease in mean systolic blood pressure occurred following regadenoson in both groups during both stress MPIs, with the largest decrease seen in Ex-Reg MPI1 (means ranged from −4 to −17 mmHg) (Fig. 4B). Systolic blood pressures <90 or ≥200 mmHg were seen in <4% of patients (Table 7).

**Table 7:** Hemodynamic effects (safety analysis set)

	Ex-Reg		Regadenoson	
Hemodynamic effects*	Stress MPI1 Regadenoson Following Exercise (*n* = 575)	Stress MPI2 Regadenoson (*n* = 544)	Stress MPI1 Regadenoson (*n* = 567)	Stress MPI2 Regadenoson (*n* = 548)
Systolic blood pressure				
<90 mmHg	11 (1.9)	18 (3.3)	22 (3.9)	14 (2.6)
Decrease >35 mmHg	167 (29.2)	34 (6.3)	54 (9.5)	38 (6.9)
≥200 mmHg	5 (0.9)	1 (0.2)	2 (0.4)	2 (0.4)
Increase ≥50 mmHg	11 (1.9)	4 (0.7)	2 (0.4)	2 (0.4)
≥180 mmHg and increase of ≥20 mmHg from baseline	28 (4.9)	13 (2.4)	10 (1.8)	6 (1.1)
Diastolic blood pressure				
<50 mmHg	17 (3.0)	12 (2.2)	17 (3.0)	17 (3.1)
Decrease >25 mmHg	35 (6.1)	22 (4.0)	30 (5.3)	28 (5.1)
≥115 mmHg	4 (0.7)	4 (0.7)	2 (0.4)	2 (0.4)
Increase ≥30 mmHg	13 (2.3)	10 (1.8)	6 (1.1)	6 (1.1)
Heart rate				
>100 BPM	187 (43.6)	128 (23.8)	170 (30.6)	134 (24.6)
Increase >40 BPM	31 (5.4)	47 (8.6)	88 (15.5)	51 (9.3)

All values are *n* (%)

Baseline was defined as immediately prior to regadenoson administration. When the baseline for Ex-Reg Stress MPI1 (Regadenoson Following Exercise) was defined as assessments immediately prior to exercise, the results were (*n*, %): systolic blood pressure <90 mmHg (11, 1.9), decrease >35 mmHg (46, 8.0), ≥200 mmHg (8, 1.4), increase ≥50 mmHg (27, 4.7), ≥180 mmHg and increase of ≥20 mmHg from baseline (43, 7.5); diastolic blood pressure <50 mmHg (16, 2.8), decrease >25 mmHg (35, 6.1), ≥115 mmHg (5, 0.9), increase ≥30 mmHg (17, 3.0); heart rate >100 BPM (308, 54.6), increase >40 BPM (225; 39.3)

*BPM,* beats per minute; *MPI,* myocardial perfusion imaging

*The denominator for each group is the number of patients who did not meet the criteria at baseline and had at least one non-missing value during treatment. For each patient, the worst case among all post-baseline measurements was used

#### Radiation Dose

Approximate mean radiation dose received for both groups was 2.8 ± 0.9 mSv during the rest scan, 8.0 ± 1.6 mSv for each stress scan, and 18.5 ± 3.9 mSv total (total range 6.5-30.5 mSv). Actual administered radiotracer dose was contemporaneously recorded.

## Discussion

Our study addresses key issues pertinent to stress MPI in patients who are ambulatory, but may not be able to attain an adequate workload on treadmill exercise—a group that constitutes a substantial proportion of patients referred for stress MPI in current practice. The study investigated the assessment of ischemic status and safety of a rapid conversion of inadequate treadmill exercise to pharmacological stress test. The primary endpoint of non-inferiority of majority agreement rate of reader self-agreement for the presence or absence of ischemia between the Ex-Reg and Regadenoson groups was met. Regadenoson administered 3 minutes post exercise during recovery does not alter the interpretation of the images from regadenoson administered at rest.

Target-to-background ratios were greater and subdiaphragmatic radiotracer interference was less frequent when regadenoson was administered 3 minutes post exercise during recovery than when regadenoson was administered at rest. This was anticipated given the increase in blood flow proportional uptake in the myocardium, whereas exercise limits or shunts activity from the abdominal organs. Improved counts, however, did not translate into clear improvement in image quality, as overall image quality was predominately excellent/good for studies in both groups.

This protocol allows patients to attempt exercise first, and then receive regadenoson at 3 minutes post exercise during recovery only if adequate stress is not achieved. This facilitates potential rapid conversion of a non-diagnostic exercise study to a pharmacologic stress study at the same visit. This approach allows stress laboratories to attempt exercise first in patients who might not need pharmacologic stress and obtain an assessment of functional capacity.^2^,^6^ The protocol was generally well tolerated and adverse events were consistent with the known safety profile of regadenoson. However, there were more patients with ischemic ST segment changes (2.8%) compared to regadenoson administered at rest (0.4% to 0.9%; see Table 4). Also, the protocol was associated with one patient who developed ACS following exercise and regadenoson and another patient who had a MI 7.5 hours following exercise and the administration of regadenoson. Upon case review, both of these Ex-Reg group patients experienced ischemic symptoms and ECG changes during exercise or recovery prior to regadenoson. In our view, these two adverse events were avoidable. A third patient developed significant ischemia during submaximal exercise testing, appropriately did not receive regadenoson, and eventually received a coronary stent. Based on these findings, clinicians supervising an exercise test in which regadenoson may be administered in recovery should carefully monitor for symptoms and ECG changes of ischemia as well as abnormal hemodynamic responses to exercise during exercise and during the 3-minute early recovery period prior to regadenoson administration. If the supervising clinician interprets ischemia as present during exercise or recovery, then radiotracer can be administered without regadenoson if the MPI is to proceed.

Previous studies in which low-level or symptom-limited exercise was combined with regadenoson found the combination to be well tolerated without serious adverse effects and associated with improved image quality.^3^,^6^,^13^ Since several prior studies have observed that increases and decreases of asymptomatic blood pressure can occur with the combination, the protocol adopted for this trial allowed for a cool down prior to regadenoson administration.^3^,^6^,^13^ Perhaps as a result of this modification, we did not observe an increase in the rate of clinically meaningful hypertension or hypotension when regadenoson was administered 3 minutes post exercise compared to regadenoson administered at rest.

Although previous studies of low-level exercise with adenosine and regadenoson have often noted more RPDs with combined exercise/vasodilator stress,^2^,^5^,^10^,^11^ in the current study the blinded readers interpreted more RPDs on MPI2 than on MPI1 in both the Ex-Reg and Regadenoson groups. This is consistent throughout the assessments of their interpretations (Tables 3 and 4 and Electronic Supplementary Material Table 1). This finding is most likely related to residual radioactivity from the resting scan generally performed on the same day as MPI1 (“shine through” of rest into stress). The second stress scan (MPI2), acquired ≥24 hours after the resting scan, would not have this residual activity as the resting counts would be negligible at ≥24 hours.^24^,^25^ This effect was anticipated to be small when the study was designed, but may be larger in clinical practice than is commonly appreciated. From a practical standpoint, this finding raises an important clinical question whether current widely used, same-day rest/stress, single-isotope studies employing the guideline-recommended 1:3 dosing ratio of technetium-99m agents may actually underestimate RPDs and whether higher isotope ratios of rest/stress (i.e., 1:4) may be needed to optimize detection of RPDs and avoid a “shine through” effect.^26^ This aspect merits further study.

### Limitations

The small number of patients with ≥2 segments with RPDs limits the ability of EXERRT to investigate changes in sensitivity for RPDs when exercise is added to regadenoson. However, the similarity in agreement rates (based on majority reader agreement) on ischemic status under the two procedures does not indicate any evidence of substantial differences in clinical conclusions. Compared to the pivotal regadenoson trials, fewer RPDs were seen in the current trial.^17^ Nuclear imaging laboratories in the United States currently report less ischemia than a decade or two ago and this trend likely played a role.^27^,^28^ The frequency of ischemia seen in our study is comparable to that reported by contemporary nuclear laboratories.^27^,^28^ In this sense, the study population was appropriate for testing the efficacy and safety of the Ex-Reg protocol.

Only one resting scan was obtained to which both stress MPIs were compared. From a practical standpoint, as there was no difference in patient clinical status during the study period, a single scan was deemed sufficient and enabled minimization of radiation exposure for study subjects.

## Conclusions

Administering regadenoson 3 minutes into recovery following inadequate exercise provides comparable categorization of segments with RPDs, appears to be well tolerated, and results in improved heart-to-liver/gut ratios. However, regadenoson should not be given immediately after exercise to patients who develop signs or symptoms of ischemia during exercise or recovery.

## New Knowledge Gained

Patients undergoing exercise MPI who do not achieve adequate exercise stress may be converted to a pharmacologic test with the administration of regadenoson at 3 minutes of recovery if a careful evaluation of symptoms, signs, and ECG do not suggest the presence of ischemia.

## Electronic supplementary material

Below is the link to the electronic supplementary material.

Supplementary material 1 (DOCX 601 kb)

Supplementary material 2 (DOCX 38 kb)

Supplementary material 3 (PPTX 600 kb)

## Abbreviations

ACS: Acute coronary syndrome
CAD: Coronary artery disease
CI: Confidence interval
ECG: Electrocardiogram
METs: Metabolic equivalents
MI: Myocardial infarction
MPHR: Maximum predicted heart rate
MPI(s): Myocardial perfusion imaging(s)
RPD(s): Reversible perfusion defect(s)
SPECT: Single photon emission computerized tomography
