# Supplementary material for: The EXERRT trial: “EXErcise to Regadenoson in Recovery Trial”: A phase 3b, open-label, parallel group, randomized, multicenter study to assess regadenoson administration following an inadequate exercise stress test as compared to regadenoson without exercise for myocardial perfusion imaging using a SPECT protocol
Source: J Nucl Cardiol. 2017 Feb 21;24(3):788–802. doi: 10.1007/s12350-017-0813-3 (PMC5491644; doi:10.1007/s12350-017-0813-3)
Supplement: Supplementary file 1 — Supplementary material 1 (DOCX 601 kb) [file 12350_2017_813_MOESM1_ESM.docx]

**Online Resource: Journal of Nuclear Cardiology**

**The EXERRT trial - “EXErcise to Regadenoson in Recovery Trial”: A phase 3b, open-label, parallel group, randomized, multicenter study to assess regadenoson administration following an inadequate exercise stress test as compared to regadenoson without exercise for myocardial perfusion imaging using a SPECT protocol**

Gregory S. Thomas, MD, MPH, MASNC,^a^ S. James Cullom, PhD,^b^ Therese M. Kitt, MD,^c^ Kathleen M. Feaheny, MS,^c^ Karthikeyan Ananthasubramaniam, MD, FASNC,^d^ Robert J. Gropler, MD, FASNC,^e^ Diwakar Jain, MD, FASNC,^f^ and Randall C. Thompson, MD, FASNC^g^

^a^ MemorialCare Heart & Vascular Institute, Long Beach Memorial Medical Center, Long Beach, CA, and University of California, Irvine, CA

^b^ AdaptivePharma, Leawood, KS, and University of Missouri, Columbia, MO

^c^ Astellas Pharma Global Development, Northbrook, IL

^d^ Department of Internal Medicine, Heart and Vascular Institute, Henry Ford Hospital, Detroit, MI

^e^ Division of Radiological Sciences, Mallinckrodt Institute of Radiology, Washington University School of Medicine, St Louis, MO

^f^ Cardiovascular Nuclear Imaging Laboratory, New York Medical College, Westchester Medical Center, Valhalla, NY

^g^ Saint Luke’s Mid America Heart Institute, Kansas City, MO, and University of Missouri-Kansas City, Kansas City, MO

**Corresponding author:** Gregory S. Thomas, MD, MPH, MASNC

Email: [gthomas1@memorialcare.org](mailto:gthomas1@memorialcare.org)

**ONLINE RESOURCE METHODS**

**Participants**

**Inclusion criteria**

Patients were eligible for the study if all of the following applied:

1. Institutional Review Board (IRB)/Independent Ethics Committee (IEC) approved written Informed Consent and privacy language as per national regulation (e.g., HIPAA Authorization for US sites) was obtained from the patient or legally authorized representative prior to any study-related procedures (including withdrawal of prohibited medication, if applicable).
2. Male and female patients ≥18 years of age referred for an exercise or pharmacologic stress test SPECT MPI procedure for the evaluation of coronary artery disease (CAD) were eligible for study participation. Patients referred for pharmacologic stress should have had a reasonable potential of attempting exercise stress in the opinion of the investigator. Patients must have had one of the following:
   1. Past ischemia on any prior imaging stress test without invasive intervention on the artery subtending this territory.
   2. Patients with known CAD who had symptoms similar to previous ischemic symptoms, or recent onset of symptoms or recently worsened symptoms suggestive of ischemia.
   3. Diamond Forrester estimated pretest probability of CAD ≥50%.
   4. History of most recent coronary artery bypass grafting (CABG) surgery or most recent percutaneous coronary intervention (PCI) >10 years (patients who were >30 days, but <10 years post CABG or PCI could be included if they met criteria a, b, or e).
   5. Previously demonstrated 100% occlusion by invasive coronary or computed tomography angiography without successful intervening revascularization.
3. Patients with CAD must have had an intermediate to low risk for immediate revascularization.
4. Female patients must have been either: of non-childbearing potential (post-menopausal [defined as women who were ˃46 years of age and last menstrual period was >2 years previously] prior to screening, or documented surgically sterile or status post hysterectomy [at least 1 month prior to screening]); or, if of childbearing potential, must have had a negative urine or serum pregnancy test at screening and prior to each imaging day, and if sexually active, must have used two forms of birth control (at least one of which must have been a barrier method) starting at screening and throughout the study period and for 28 days after the final study drug administration.
5. Female patients must not have been breastfeeding at screening or during the study period, and for 28 days after the final study drug administration.
6. Female patients must not have donated ova starting at screening and throughout the study period, and for 28 days after the final study drug administration.
7. Patients must have abstained from eating and drinking (except water) 3 hours prior and 30 minutes post study drug administration. The prohibition against eating may have been adjusted for patients with diabetes based on investigator judgment.
8. Patients must have abstained from smoking 3 hours prior to each study drug administration.
9. Patients must have abstained from any intake of methylxanthine (caffeine)-containing foods and beverages, such as coffee, colas, or chocolate ≥12 hours prior to administration of study drug, as these foods may alter regadenoson effects.
10. Patients were able to safely abstain from dipyridamole or dipyridamole-containing compounds or xanthine bronchodilators (aminophylline/theophylline) in the last 48 hours prior to study drug administration.
11. Patients agreed not to participate in another interventional study while on treatment.

**Exclusion criteria**

Patients were excluded from participation if any of the following applied:

1. Patients were concurrently participating in another drug study or had received an investigational drug within 30 days prior to screening.
2. Patients had a clinically significant illness, medical condition, or laboratory abnormality, active or historic, which in the Investigator's opinion precluded participation in the study.
3. During the performance of exercise, in the investigator’s judgment, patients achieved ≥85% maximum predicted heart rate (MPHR) and ≥5 metabolic equivalents (METs) or in the investigator’s judgment, significant ischemia was present and pharmacological stress was not required.
4. Female patients who were pregnant or lactating.
5. Patients on dialysis for end stage renal disease or had a history of glomerular filtration rate (GFR) <15 mL/min (calculated using Modification of Diet in Renal Disease formula).
6. Patients had a history of coronary revascularization by either PCI or CABG within 1 month prior to the rest MPI.
7. Patients had a pacemaker or an implantable cardioverter defibrillator.
8. Patients were referred for exercise MPI and had a Duke Activity Status Index Score of ˃4.7 METs unless currently taking beta blockers or rate-slowing calcium channel blockers, or had known chronotropic incompetence.
9. Patients had a history of acute myocardial infarction (MI) or high-risk unstable angina within 30 days prior to the rest MPI or had prior cardiac transplantation.
10. Patients had required a change in medications that may have affected the rate-pressure product within 5 days of the rest MPI. Change was defined as an addition, discontinuation, or dose change of any medication (e.g., beta blocker, calcium channel blocker, antihypertensive, or long-acting nitrate) that could alter the rate-pressure product.
11. Patients had uncontrolled hypertension at any point during stress MPI1 prior to exercise testing (i.e., systolic blood pressure [SBP] ≥180 or diastolic blood pressure [DBP] ≥95 mm Hg on two consecutive measurements while at rest).
12. Patients had severe aortic stenosis or hypertrophic cardiomyopathy with obstruction or had decompensated congestive heart failure within 30 days of baseline period.
13. Patients had left bundle branch block, uncontrolled atrial fibrillation (i.e., heart rate >100 BPM), or significant ventricular arrhythmias or had a history of second- or third-degree heart block or significant sinus node dysfunction.
14. Patients required emergent cardiac medical intervention or catheterization.
15. Patients had a history of severe respiratory disease including: asthma, chronic obstructive pulmonary disease, or other bronchospastic reactive airway disease or who were on 24-hour continuous oxygen.
16. Patients had symptomatic hypotension (temporary and reversible conditions that no longer existed were allowed) or patients had an SBP <90 mm Hg during MPI1 immediately prior to exercise testing.
17. Patients were allergic or intolerant to aminophylline.
18. Patients were allergic or intolerant to regadenoson or any of its excipients.
19. Patients were unable or unwilling to comply with the procedure schedule.
20. Patients had been previously enrolled in this study protocol or any previous regadenoson study sponsored by Astellas.

**Stress Testing and Imaging**

All patients were imaged with either a 1-day (96%, 1,102/1,142 patients) or 2-day rest-stress ^99m^Tc-sestamibi or ^99m^Tc-tetrofosmin SPECT MPI protocol without attenuation correction.^1^ Following a baseline visit, patients underwent a resting SPECT MPI. Patients then initiated exercise using a standard or modified Bruce protocol. If the patient achieved ≥85% of MPHR and ≥5 METs of activity, the patient was discontinued from the study. For all participants, MET levels were estimated and not measured directly. If the patient did not achieve ≥85% of MPHR and/or ≥5 METs of activity and did not meet other discontinuation criteria, they transitioned into a 3- to 5-minute walking recovery. Randomization was performed using an opaque envelope containing the group assignment. The envelope was opened once randomization criteria were met.

During the first 3 minutes of recovery, each patient was randomly assigned in a 1:1 ratio into Ex-Reg or Regadenoson (Figure 1, full article). Sites were requested to make a best effort to reproduce operator-dependent parameters such as detector orbit and patient positioning between scans. Radiotracer doses at stress were required to be within ± 10% of those in repeat imaging. Immediately following each of the two stress SPECT acquisitions, a single 60-second planar anterior image was acquired to evaluate the impact of extra-cardiac activity on image quality.

Stress-rest image sets were created by pairing the initial rest image with the stress image obtained with regadenoson after exercise to create MPI1 of Ex-Reg and the stress image obtained with regadenoson to create MPI1 of Regadenoson. MPI2 in both groups was created using the stress SPECT image performed after regadenoson administration at rest 1-14 days later with the initial rest image. The independent image reviewers underwent training prior to reading any of the study images. Test cases were read independently, individual assessments reviewed as a group, and agreement obtained. Each reviewer read the images independently. Images from each patient were read on three separate occasions with at least 10 business days between each read. The first session consisted of either rest/stress MPI1 or rest/stress MPI2 presented in random order. Session 2 consisted of the rest/stress MPI not presented during the first session. The single rest image was locked and paired with the second stress image set for all interpretations. Session 3 consisted of reading planar images and stress MPI1 and MPI2 (i.e., side-by-side comparison).

**Blinding and read preparation**

All image data were de-identified for site, patient, and imaging sequence and reviewed for quality control, technical errors, and compliance with study protocols by the imaging core laboratory. Images with unacceptable quality (i.e., determined to be of poor quality by an individual reader) were not evaluated by that reader. Images were reviewed in cine format for detection and application of motion correction as needed. All rest/stress perfusion and electrocardiogram (ECG)-gated projections were filtered prior to reconstruction using the Butterworth filter (critical frequency 0.7, order 6). Transverse images were then reconstructed using the filtered-back projection algorithm as provided in the Emory Cardiac Toolbox, V3.2 and reoriented to the short, horizontal and vertical long axis views for blinded expert evaluation and normal database quantitation.

**Planar imaging**

The average number of counts per pixel was computed for each of the following three regions of interest (ROIs) from the stress planar acquisitions in MPI1 and MPI2: whole heart, right upper quadrant of the liver, and a 5 x 5 pixel region centered 5 pixels below the inferior wall. From these values, heart:liver, heart:gut and heart:liver + gut ratios were computed.

**Blinded read process**

Image evaluation was performed on two identically configured computer systems at the imaging core laboratory. Each reader evaluated all of the images for patients who completed all three scans (rest, MPI1, and MPI2). None of the readers were affiliated with an investigator site, the study sponsor, or the core imaging laboratory. Prior to the read, readers received uniform training on the rules for image evaluation, equipment operation, case report form completion, and regulatory compliance. Readers were permitted to adjust stress/rest image orientation, myocardial boundary contours, and color table intensity.

All readers followed a systematic review process as follows: overall diagnostic quality was evaluated including presence of technical or patient-related errors or artifacts, identifying the type of abnormality when possible. If diaphragmatic artifacts were identified, the reader recorded the segments affected and rated the impact on a 4-point scale: None, Slight, Moderate, or Severe. A “Primary SPECT Review” was then performed for extent and severity of perfusion defects for each myocardial segment employing a 5-point scale for stress and rest images. A “Secondary Review” was then performed to assess the variables for the remaining secondary variables. Segmental scoring was not performed if diagnostic quality was determined to be inadequate (poor). However, if the overall study was evaluated as adequate, but individual segments non-evaluable, the remaining segments were analyzed. After the Secondary Review, the readers conducted a side-by-side assessment of the MPI1 and MPI2 stress/rest image sets and evaluated ischemic extent and image quality due to sub-diaphragmatic interference.

**SPECT image evaluation**

Overall quality of the SPECT scans was rated by reviewers as: Excellent, high-quality images, unequivocally adequate for segmental scoring; Good, images that were not quite excellent but the quality was adequate for segmental scoring; Fair, images that were not of good quality but were still of high enough quality for segmental scoring and; Poor, images were not considered interpretable. If any of the rest or stress image sets was graded as poor by the reader, segmental scoring was not permitted and the patient was not included in the efficacy analysis set. Reasons for poor image quality included patient motion, breast attenuation, diaphragmatic attenuation, reconstruction artifacts, inadequate myocardial statistics, incomplete exam, incomplete anatomical coverage, gastrointestinal activity, or other.

**Criteria for Trial Discontinuation**

Criteria for discontinuation from the trial during exercise testing were modified during the study as defined in the full article. The protocol was amended following a report of a patient experiencing acute coronary syndrome during the study. The protocol was amended such that if patients exhibited signs or symptoms of ischemia during exercise or recovery, exercise testing was discontinued and the patient terminated from the study. Details of the discontinuation criteria were:

- Patient experienced a serious or intolerable adverse event.
- In the investigator’s opinion, patient was non-compliant with the protocol requirements.
- Patient’s health would have been jeopardized by continued participation.
- Patient wished to withdraw consent.

If the patient experienced any of the following American College of Cardiology/ American Heart Association 2002 guidelines^2^ for absolute indication for terminating exercise testing, exercise testing was to be discontinued and the patient discontinued from the study:

- Drop in SBP >10 mm Hg from baseline blood pressure despite an increase in workload, when accompanied by other evidence of ischemia
- Moderate to severe angina
- Increasing nervous system symptoms (e.g., ataxia, dizziness, or near syncope)
- Signs of poor perfusion (cyanosis or pallor)
- Technical difficulties in monitoring ECG or SBP
- Sustained ventricular tachycardia
- ST elevation (≥1.0 mm) in leads without diagnostic Q-waves (other than V1 or a VR)

Patients with signs or symptoms suggestive of myocardial ischemia during exercise or recovery were not to receive regadenoson in conjunction with the exercise stress test. If the patient experienced any of the following during exercise testing or during recovery prior to the administration of regadenoson, the patient was discontinued from the study and regadenoson was not administered:

- Moderate to severe angina
- Increasing chest pain
- Pain consistent with angina and/or suspicious of ischemia or pain in the chest, arm, neck, or jaw for ≥10 seconds
- Pain in the chest, arm, neck, or jaw with ECG changes of ischemia (such as horizontal or downsloping ST depression ≥1 mm with or without T-wave abnormalities) that occurs during exercise or recovery
- Increasing nervous system symptoms (e.g., ataxia, dizziness, or near syncope)
- Signs of poor perfusion (cyanosis or pallor)
- Technical difficulties monitoring ECG or SBP
- Sustained ventricular tachycardia
- ST elevation (≥1.0 mm) in leads without diagnostic Q waves (other than V1 or a VR)
- ECG changes occurring during exercise or recovery without angina/and or symptoms suspicious of ischemia that the investigator interpreted as clearly diagnostic of ischemia, including >1 mm of horizontal or downsloping ST depression or ischemic evolution during recovery or marked axis shift. (Note: an abnormal resting ECG may have impacted the investigator’s interpretation of changes during exercise and/or recovery)
- Arrhythmias such as triplets of premature ventricular contractions, symptomatic supraventricular tachycardia or supraventricular tachycardia lasting >10 seconds, second-degree heart block, or symptomatic bradyarrhythmias
- Development of bundle branch block or intraventricular conduction defect (IVCD) that could not be distinguished from ventricular tachycardia
- Hypertensive response: SBP >250 mm Hg and /or DBP >115 mm Hg
- Any other sign or symptom that in the medical opinion of the investigator represented a manifestation of myocardial ischemia

If the patient experienced any of the following clinically significant adverse events during the initial regadenoson injection, the patient was discontinued from study participation and did not undergo the second day of imaging procedures. The events included but were not limited to:

- High-degree atrioventricular (AV) block in the absence of a pacemaker
- Syncope
- Persistently high blood pressure (SBP >180 mm Hg or DBP of ≥95 mm Hg on two consecutive measurements)
- Clinical signs of hypotension
- Unstable angina

**Endpoints/Statistical Methodology**

**Analysis variables**

The 17-segment model was utilized. The number of reversible “ischemic” defects (reversible perfusion defects [RPDs]) was counted to determine if there was a change in the number of reversible defects categorized as absence (0-1) or presence (≥2) of ischemia between SPECT MPI1 and MPI2 as assessed by each of the three blinded independent expert readers. The number of reversible defects was calculated as the number of segments with positive (stress score – rest score) differences where the segment was not counted if the stress score was less than 2. Segments were counted as having a RPD if the stress score was greater than the rest score and the stress score was ≥2, using the “natural” pairing (i.e., initial stress with initial rest and second stress with initial rest).

Scoring involved points assigned to each segment in direct proportion to the perceived count density of the segment based on a 5-point scale for radiopharmaceutical uptake:

*Category Score*

0 = normal radiotracer uptake

1 = slightly reduced radiotracer uptake

2 = moderately reduced radiotracer uptake

3 = severely reduced radiotracer uptake

4 = absent radiotracer uptake

The Summed Stress Score (SSS) was calculated as the sum of the stress scores across the 17 segments. The Summed Rest Score (SRS) was calculated as the sum of the rest scores across the 17 segments. The Summed Difference Score (SDS) was calculated as the difference in the SSS and SRS (SSS – SRS).

Efficacy Endpoints

Primary efficacy endpoint

The primary endpoint was the binary outcome: Majority Agreement Yes or Majority Agreement No and is described in the full article.

Secondary efficacy endpoints

The secondary efficacy endpoints included the following:

- Agreement rates between SPECT imaging with regadenoson following inadequate exercise stress testing and SPECT imaging with regadenoson alone using three categories for ischemia (0-1, 2-4, ≥5 reversible segments) and two categories for ischemia (0-1, ≥2 reversible segments) based on the median count of the number of reversible defects across the three blinded independent expert readers.
- Agreement of image pairs with regard to reader SDS and SSS, and a paired (side-by-side) comparison of ischemic extent.
- Overall assessment of image quality defined by the independent readers as Excellent, Good, Fair, or Poor.
- Target (heart)-to-background radiotracer ratio of the heart to liver and heart to gut.
- Target (heart)-to-background radiotracer ratio of the combined background ratio of gut and liver (mean of gut and liver).
- Sub-diaphragmatic radiotracer activity interference with cardiac image quality using a 4-point scale (0 = none; 1 = slight; 2 = moderate; 3 = severe).

*Calculations to determine target-to-background radiotracer ratios:*

The ICON imaging specialist drew ROIs corresponding to heart, liver, and gut. Each reader was queried as to whether the ROIs drawn were acceptable. The reader answered “Yes” or “No.” If the reader answered No, the reader had the opportunity to adjust the ROIs. If planar data were not available or too poor to assess, the reader recorded the data “Not Available.” The imaging software measured the pixel activity counts and the pixel area of the circled region

where ROI was either heart, liver, or gut.

The imaging database provided the computation of the above ratio along with the computation of three different variations of radiotracer uptake ratios:

1. Heart-to-liver radiotracer uptake ratio =

$$\frac{Average Pixel Activity in Heart ROI}{Average Pixel Activity in Liver ROI}$$

2. Heart-to-gut radiotracer uptake ratio =

$$\frac{Average Pixel Activity in Heart ROI}{Average Pixel Activity in Gut ROI}$$

3. Heart-to-(gut and liver) – since the pixel area of the final liver and gut ROIs were not necessarily equal, a weighted average was computed:

Heart-to-(gut and liver) radiotracer uptake ratio =

$\frac{Average Pixel Activity in Heart ROI}{\left( \frac{Pixel Area Liver ROI*Pixel Activity Count Liver ROI+Pixel Area Gut ROI*Pixel Activity Count Gut ROI}{Pixel Area Liver ROI+Pixel Area Gut ROI} \right)}$

Safety variables

The safety composite variable was defined as the percentage of patients who experienced at least one treatment emergent clinically significant cardiac event.

A clinically significant cardiac event was defined as:

- Any of the following events found on the Holter ECG/12-lead ECG within 1 hour after regadenoson administration:
  - Ventricular arrhythmias (sustained ventricular tachycardia, ventricular fibrillation, torsade de pointes, ventricular flutter),
  - ST-T depression ($\geq$2 mm),
  - ST-T elevation (≥1 mm),
  - AV block (2:1 AV block, AV Mobitz I, AV Mobitz II, complete heart block)
  - Sinus arrest >3 seconds

Or

- Treatment-emergent adverse event (TEAE) per the Medical Dictionary for Regulatory Activities (MedDRA): Standardized MedDRA Queries (Narrow Scope) for myocardial infarction (Included MedDRA Preferred Terms of Acute coronary syndrome, Acute myocardial infarction, Blood creatine phosphokinase MB abnormal, Blood creatine phosphokinase MB increased, Coronary artery embolism, Coronary artery occlusion, Coronary artery re-occlusion, Coronary artery thrombosis, Coronary bypass thrombosis, Kounis syndrome, Myocardial infarction, Myocardial reperfusion injury, Myocardial stunning, Papillary muscle infarction, Post procedural myocardial infarction, Post infarction angina, Silent myocardial infarction, Troponin I increased, Troponin increased, Troponin T increased)

Or

- TEAE preferred term of angina unstable within 24 hours of regadenoson administration

Safety was also assessed by evaluation of the following variables:

- TEAEs
- Vital signs
- 12-lead ECG
- Radiation exposure

TEAE: an adverse event observed from the time of administration of regadenoson to within 24 hours of administration of study drug within each visit.

Radiation from SPECT was collected in mCi for rest and stress scans. The rest, stress, and sum of the rest and stress doses were multiplied by 0.23 for ^99m^Tc tetrofosmin and by 0.285 for ^99m^Tc sestamibi to obtain the radiation dose expressed in mSv.^3^

STATISTICAL METHODOLOGY

General Considerations

For continuous variables, descriptive statistics included the number of patients (n), mean, standard deviation, median, minimum, and maximum. Frequencies and percentages were displayed for categorical data.

All statistical comparisons were made using two-sided tests at the α=0.05 significance level unless specifically stated otherwise. All null hypotheses were of no treatment difference; all alternative hypotheses were two-sided, unless specifically stated otherwise.

Study Population

Demographic and other baseline characteristics

Demographic and other baseline characteristics were summarized by descriptive statistics.

Targeted medical history and whether the patient was referred for pharmacologic stress test or exercise stress test or both were also summarized by treatment group.

Exercise protocol (Bruce protocol, modified Bruce protocol), percent MPHR and maximum METs achieved were summarized. Post hoc summaries were done for duration of exercise on the treadmill. Percent MPHR and maximum METs achieved by exercise protocol (Bruce protocol, modified Bruce protocol) were summarized by treatment group. Percent MPHR = maximum heart rate/(220-age)×100. Maximum heart rate was the highest heart rate (i.e., pulse rate) achieved during exercise including the 3-minute walk recovery (prior to study drug administration).

Analysis of Efficacy

Analysis of primary endpoint

A description of the analysis of the primary endpoint is provided in the full article.

Analysis of secondary endpoints

All secondary efficacy assessments were performed for the efficacy analysis set population.

Agreement rates – using two and three categories for ischemia

The secondary variables including the median count (across the three readers) of the number of segments with reversible defects categorized as absence (0-1) or presence (≥2) of ischemia and categorized using three categories for ischemia (0-1, 2-4, ≥5 reversible segments) were assessed.

The sample size of 450 evaluable patients in each group for a total of 900 patients completing all three planned images using a one-sided alpha level of 0.025 and a non-inferiority margin of 0.10 provided 85% power for the agreement rate using two categories of ischemia variable. The estimated inter-test agreement for SPECT without exercise was estimated to be 0.78. This was the observed rate from the regadenoson pivotal studies when using the dichotomous classification of presence or absence. The non-inferiority margin of 0.10 was consistent with the margin used in the pivotal studies when implementing a dichotomous classification and represents a clinically meaningful difference.^4,5^

The analysis for 2 categories for ischemia used the agreement rate of the absence or presence of ischemia for successive SPECT images with regadenoson in Ex-Reg and the agreement rate for successive SPECT images with regadenoson in Regadenoson. The agreement rate for two images was calculated as 0.5 (A / (A+B)) + 0.5 (D / (C+D)) where A was defined as the count of patients whose ischemia status was assessed as absent at both stress MPI1 and MPI2, B was defined as the count of patients whose ischemia status was assessed as present at stress MPI1 and absent at stress MPI2, C was defined as the count of patients whose ischemia status was assessed as absent at stress MPI1 and present at stress MPI2, and D was defined as the count of patients whose ischemia status was assessed as present at both stress MPI1 and stress MPI2.

The analysis for the three categories of ischemia was similar to the analysis for the two categories with the agreement rate calculated as 0.333 [(a/(a+b+c)) + (e/(d+e+f)) + (i/(g+h+i))] where patient counts a, b, c, d, e, f, g, h, and i were defined as follows:

a = count of patients assessed with 0-1 RPD at stress MPI1 and MPI2,

b = count of patients assessed with 2-4 RPDs at stress MPI1 and 0-1 RPD at MPI2,

c = count of patients assessed with ≥5 RPDs at stress MPI1 and 0-1 RPD at MPI2,

d = count of patients assessed with 0-1 RPD at stress MPI1 and 2-4 RPDs at MPI2,

e = count of patients assessed with 2-4 RPDs at stress MPI1 and MPI2,

f = count of patients assessed with ≥5 RPDs at stress MPI1 and 2-4 RPDs at MPI2,

g = count of patients assessed with 0-1 RPD at stress MPI1 and ≥5 RPDs at MPI2,

h = count of patients assessed with 2-4 RPDs at stress MPI1 and ≥5 RPDs at MPI2,

i = count of patients assessed with ≥5 RPDs at stress MPI1 and MPI2

An assessment of non-inferiority was provided by a confidence interval (CI) on the difference in agreement rates (agreement rate for Ex-Reg minus the agreement rate for Regadenoson) for the two- and three-category variables. For the two-category variable: the lower confidence bound of the one-sided alpha level of 0.025 of the difference in agreement rates must have exceeded -0.10 in order to demonstrate non-inferiority. For the three-category variable: the lower confidence bound of the one-sided alpha level of 0.025 of the difference in agreement rates must have exceeded –0.133 in order to demonstrate non-inferiority. The non-inferiority margin of –0.133 was consistent with the margin used in the pivotal studies when implementing a three-category classification.^4,5^

**Secondary efficacy variable: agreement of image pairs – SDS and SSS**

Each reader’s SSS and SDS were calculated and the mean value (rounded to the nearest integer) across the three readers was computed. The SSS was classified into 4 group categorical variables based on the score: 0-3, 4-7, 8-11, and ≥ 12. The SDS was classified into 3 group categorical variables: 0–6, 7–13, and ≥14. The SDS was also classified into 4 group categorical variables: 0-2, 3-6, 7-13, and ≥ 14 for a post hoc summary. For SDS, agreement within each group (Ex-Reg: agreement between regadenoson following exercise and regadenoson; Regadenoson: agreement between regadenoson and regadenoson) was assessed by Cohen’s Kappa statistic and Cohen’s weighted Kappa statistic.

For SSS, agreement rates were calculated for each category within each group. The difference in the total agreement rates between the groups and associated 95% CI based on the normal approximation was calculated.

**Secondary efficacy variable: agreement of image pairs – paired (side-by-side) comparison of ischemic extent**

Each reader evaluated the initial stress SPECT MPI scan compared to the patient’s second stress SPECT MPI scan (blinded at time of the evaluation) for whether there was Less, Same, or More RPDs. The median of the assessment of the three readers was calculated. Within each group, a sign test was performed to test if exercise had no effect or had an effect on evaluating RPDs.

**Secondary efficacy variables: overall assessment of image quality (Excellent, Good, Fair, or Poor)**

Overall image quality was rated by the blinded readers as Excellent, Good, Fair, or Poor. The distribution of images across these four categories was summarized by group by SPECT MPI scan (rest, initial stress, second stress) by reader. The median rating across the three readers was also summarized with 1 = Poor, 2 = Fair, 3 = Good, and 4 = Excellent.

**Secondary efficacy variables: Target (heart)-to-background radiotracer ratio of the heart to liver, heart to gut, and heart to combined gut and liver (mean of gut and liver)**

The ratios were summarized descriptively by group by stress SPECT MPI. Within each group, the median uptake ratio difference and CI between SPECT MPI scans (initial stress SPECT MPI and second stress SPECT MPI) was estimated using Hodges-Lehman estimate.

For Ex-Reg, the superiority of regadenoson following exercise image quality was assessed by comparing regadenoson following exercise (stress MPI1) and regadenoson (stress MPI2) using Wilcoxon signed rank test.

**Secondary efficacy variables: sub-diaphragmatic radiotracer activity interference with cardiac image quality using a 4-point scale**

Each reader assessed the sub-diaphragmatic radiotracer interference with cardiac image quality using a 4-point scale of none, slight, moderate, or severe for the stress SPECT MPI scan data only. The median rating across the three readers was used. For each treatment group, a 4x4 table showing frequencies of each pair rating of the stress scans was generated with the marginal totals. First scan marginal distributions were compared. Specifically, the average rating on a scale of 0 = none to 3 = severe and its estimated standard error was shown for each treatment group along with the regadenoson following exercise (Ex-Reg Stress MPI1) versus regadenoson alone (Regadenoson Stress MPI1) difference in average ratings and its estimated standard error. Average ratings for regadenoson following exercise (Ex-Reg Stress MPI1) and for regadenoson alone (Regadenoson Stress MPI1) were compared using an exact Cochran-Mantel-Haenszel test.

Analysis of Safety

All safety summaries were performed on the safety analysis set population.

Safety composite variable

The safety composite variable was defined as the percentage of patients who experienced at least one treatment emergent clinically significant cardiac event as previously defined. Descriptive summaries including number and percentage of patients were presented for each event and overall.

The following events were reported from the Holter (continuous) ECG data: ventricular arrhythmias (sustained ventricular tachycardia, ventricular fibrillation, torsade de pointes, ventricular flutter), AV block (2:1 AV block, AV Mobitz I, AV Mobitz II, complete heart block), and sinus arrest >3 seconds in duration. Findings of ST-T depression (≥2 mm) and ST-T elevation (≥1 mm) were reported from the 12-lead ECG data.

Adverse events

The coding dictionary for this study was MedDRA.

The number and percentage of patients were summarized for patients with any treatment-emergent adverse event (TEAE), drug-related TEAE, serious TEAE, TEAE leading to permanent discontinuation of study drug, deaths and common TEAEs that equal or exceed a threshold of 5.0% in any treatment group.

Vital signs

Baseline for vital sign assessments was defined as the last observation immediately prior to study drug administration. Additional summaries were done for Ex-Reg MPI1 (regadenoson following exercise) defining baseline as the last observation immediately prior to exercise.

Vital signs were summarized using mean, standard deviation, minimum, maximum, and median by scheduled assessment time.

The number and percentage of patients was summarized for the following potentially clinically significant vital sign criteria:

- SBP: decrease of >35 mm Hg from baseline, <90 mm Hg, ≥200 mm Hg, ≥180 mm Hg with an increase of ≥20 mm Hg from baseline, increase ≥50 mm Hg from baseline
- DBP: <50 mm Hg, decrease >25 mm Hg from baseline, ≥115 mm Hg, increase ≥30 mm Hg from baseline
- Heart rate: >100 BPM, increase >40 BPM from baseline

Other safety-related observations: radiation exposure

Radiation from SPECT was collected in mCi for rest and stress scans. Total radiation dose was the sum of the doses multiplied by 0.23 for tetrofosmin and 0.285 for sestamibi (mSv). Rest, stress, and total radiation dose received by patients was summarized by treatment group by visit and overall.^3^

**REFERENCES**

**1.** Henzlova MJ, Cerqueira MD, Hansen CL, Taillefer R, Yao SS. ASNC imaging guidelines for nuclear cardiology procedures: stress protocols and tracers. J Nucl Cardiol 2009;16:10.1007/s12350-009-9062-4.

**2.** Gibbons RJ, Balady GJ, Bricker JT, Chaitman BR, Fletcher GF, Froelicher VF, et al. ACC/AHA 2002 guideline update for exercise testing: summary article: a report of the American College of Cardiology/American Heart Association Task Force on Practice Guidelines (Committee to Update the 1997 Exercise Testing Guidelines). J Am Coll Cardiol 2002;40:1531-40.

**3.** Einstein AJ, Moser KW, Thompson RC, Cerqueira MD, Henzlova MJ. Radiation dose to patients from cardiac diagnostic imaging. Circulation 2007;116:1290-1305.

**4.** Iskandrian AE, Bateman TM, Belardinelli L, Blackburn B, Cerqueira MD, Hendel RC, et al. Adenosine versus regadenoson comparative evaluation in myocardial perfusion imaging: results of the ADVANCE phase 3 multicenter international trial. J Nucl Cardiol 2007;14:645-58.

**5.** Cerqueira MD, Nguyen P, Staehr P, Underwood SR, Iskandrian AE, ADVANCE-MPI Trial Investigators (2008). Effects of age, gender, obesity, and diabetes on the efficacy and safety of the selective A2A agonist regadenoson versus adenosine in myocardial perfusion imaging. JACC Cardiovasc Imaging 2008;1:307-16.

**ONLINE RESOURCE ACKNOWLEDGMENTS**

**Blinded Reviewers**

Richard T. George, MD, The Johns Hopkins Hospital, Baltimore, MD

Christopher L. Hansen, MD, Thomas Jefferson University, Philadelphia, PA

L. Samuel Wann, MD, Columbia St Mary’s, Milwaukee, WI

**Clinical Investigators**

**Argentina**

Fernando F. Faccio (Sanatorio San Geronimo)

Adolfo Facello (Instituto Oulton)

Anibal A. Mele (Instituto de Cardiologia la Plata)

**Peru**

David G. Galvez Caballero (Instituto Nacional Cardiovascular de EsSaludM)

**United States**

Olakunle O. Akinboboye (Laurelton Heart Specialist P.C.)

Karthikeyan Ananthasubramaniam (Henry Ford Hospital)

Ahmad A. Aslam (West Houston Area Clinical Trial Consultants, LLC)

Brad A. Bart (Hennepin County Medical Center)

Timothy Bateman (Cardiovascular Imaging Technologies)

Gholam R. Berenji (VA West Los Angeles Medical Center)

Daniel S. Berman (Cedars-Sinai Medical Center)

Stephen Bloom (Midwest Cardiology Associates)

Sabahat Bokhari (Columbia University Medical Center)

Jamieson Bourque (University of Virginia)

Christopher S. Brown (Mobile Heart Specialists, PC)

Matthew J. Budoff (Los Angeles Biomedical Research Institute)

William S. Carroll (Cardiology Associates of North America)

Arthur B. Chandler Jr. (University Cardiology Associates, LLC)

Julius Dean (St. Luke’s Cardiology Associates)

Steven Edell (Delaware Clinical Trials, LP.)

James M. Feldman (Katy Cardiology Associates)

Alvaro Gomez (Cardiovascular Research Center of South Florida)

Tauqir Y. Goraya (Michigan Heart, PC)

Robert J. Gropler (Washington University School of Medicine)

Khiet C. Hoang (Long Beach Memorial Medical Center)

John J. Hunter (Santa Rosa Cardiology Medical Group)

Diwakar Jain (Westchester Medical Center)

Arthur J. Labovitz (St. John's Mercy Medical Center)

Justin B. Lundbye (Harford Hospital)

Mauricio E. Melhado (Cardiology Partners Clinical Research Institute)

Michael I. Miyamoto (Mission Internal Medical Group)

Bharat R. Mocherla (Las Vegas Radiology)

Ronald J. Polinsky, Jr. (Berks Cardiologists, Ltd.)

Patrick J. Reddy (Watson Clinic)

John F. Schmedtje, Jr. (Roanoke Heart)

Harry M. Serfer (Elite Research and Clinical Trials, LLC)

Albert J. Sinusas (Yale University School of Medicine)

Alon A. Steinberg (Ventura Clinical Trial)

Joseph B. Thibodeau (Alegent Health Research Center)

Georgianne M. Valli-Harwood (Berkshire Medical Center)

Robert J. Weiss (Maine Research Associates)

Tsunehiro Yasuda (Massachusetts General Hospital)

Mark Zhuk (Elite Research and Clinical Trials, LLC)

**Online Resource Table 1.** Summed Difference Scores

| **Summed Difference Scores** | | | | | |
| --- | --- | --- | --- | --- | --- |
|  | **MPI2 Mean SDS** | | | **Kappa*** | **Weighted Kappa*** |
| **MPI1 Mean SDS** | 0‒6 | 7‒13 | ≥14 |
| Ex-Reg | | | | | |
| 0‒6 | 531 | 4 | 0 | 0.597 | 0.597 |
| 7‒13 | 0 | 3 | 0 |
| ≥14 | 0 | 0 | 0 |
| Regadenoson | | | | | |
| 0‒6 | 527 | 3 | 0 | 0.541 | 0.541 |
| 7‒13 | 2 | 3 | 0 |
| ≥14 | 0 | 0 | 0 |

*Cohen’s Kappa and weighted Kappa statistics

MPI, myocardial perfusion imaging; SDS, Summed Difference Score

**Online Resource Table 2.** Overall image quality

| **Image quality, *n* (%)** | **Ex-Reg**  **(*n* = 538)** | | | **Regadenoson**  **(*n* = 535)** | | |
| --- | --- | --- | --- | --- | --- | --- |
|  | **MPI1** | **MPI2** | **Rest** | **MPI1** | **MPI2** | **Rest** |
| Excellent | 224 (41.6) | 192 (35.7) | 140 (26.0) | 211 (39.4) | 205 (38.3) | 157 (29.3) |
| Good | 287 (53.3) | 303 (56.3) | 307 (57.1) | 291 (54.4) | 293 (54.8) | 308 (57.6) |
| Fair | 27 (5.0) | 43 (8.0) | 91 (16.9) | 33 (6.2) | 37 (6.9) | 70 (13.1) |

Median rating across the three readers is presented.

There were 17 patients (17/1142, 1.5%) in the safety analysis set who were omitted from the efficacy analysis set because of uninterpretable scans. Of these, three patients (Ex-Reg *n* = 1, Regadenoson *n* = 2) had scans that could not be processed by the core lab and 14 patients had at least one of three scans (rest, MPI1, MPI2) rated as poor by at least two of three readers (Ex‑Reg, *n* = 5 [4 rest, 2 MPI1, 0 MPI2]; Regadenoson, *n* = 9 [7 rest, 5 MPI1, 3 MPI2]).

MPI, myocardial perfusion imaging

**Online Resource Table 3.** Sub-diaphragmatic interference with cardiac image quality

| **Stress MPI1** | **Stress MPI2** | | | | | | | | |  | Difference,  MPI1–MPI2 (SE) |
| --- | --- | --- | --- | --- | --- | --- | --- | --- | --- | --- | --- |
|  | 0 – none | | 1 – slight | | 2 – moderate | | 3 – severe | | All (%) | Average Rating MPI1 (SE) |  |
| Ex-Reg* | | | | | | | | | | | |
| 0 – none | 0 | 1 | | 0 | | 0 | | 1(<1) | | 1.3 (0.03) | –0.1 (0.04)  Fleiss-Everitt *P*=0.019^†^ |
| 1 – slight | 0 | 141 | | 63 | | 0 | | 204 (72) | |  |  |
| 2 – moderate | 0 | 39 | | 39 | | 0 | | 78 (28) | |  |  |
| 3 – severe | 0 | 0 | | 1 | | 0 | | 1 (<1) | |  |  |
| All (%) | 0 | 181 (64) | | 103 (36) | | 0 | | 284 (100) | |  |  |
| Average Rating MPI2 (SE) | 1.4 (0.03) | | | | | | | | |  |  |
| Regadenoson* | | | | | | | | | | | |
| 0 – none | 0 | 0 | | 0 | | 0 | | 0 | | 1.3 (0.03) | ‒0.0 (0.04)  Fleiss-Everitt  *P*=0.921^†‡^ |
| 1 – slight | 0 | 133 | | 51 | | 0 | | 184 (68) | |  |  |
| 2 – moderate | 0 | 49 | | 36 | | 0 | | 85 (32) | |  |  |
| 3 – severe | 0 | 0 | | 1 | | 0 | | 1 (<1) | |  |  |
| All (%) | 0 | 182 (67) | | 88 (33) | | 0 | | 270 (100) | |  |  |
| Average MPI2 (SE) | 1.3 (0.03) | | | | | | | | |  |  |
| Ex-Reg MPI1 average – Regadenoson MPI1 average –0.0 (0.04) ^‡^ | | | | | | | | | | | |

The differences were calculated as average rating of MPI1 – MPI2 within each group. *Values are median ratings across the three readers (with or without percentages). ^†^Fleiss-Everitt *P*-value for testing Stress MPI1 = Stress MPI2 mean score. ^‡^*P*-value (CMH for testing Ex-Reg MPI1 mean rating = Regadenoson MPI1 mean rating) = 0.333.

MPI, myocardial perfusion imaging; SE, standard error
