# Supplementary material for: The EXERRT trial: “EXErcise to Regadenoson in Recovery Trial”: A phase 3b, open-label, parallel group, randomized, multicenter study to assess regadenoson administration following an inadequate exercise stress test as compared to regadenoson without exercise for myocardial perfusion imaging using a SPECT protocol
Source: J Nucl Cardiol. 2017 Feb 21;24(3):788–802. doi: 10.1007/s12350-017-0813-3 (PMC5491644; doi:10.1007/s12350-017-0813-3)
Supplement: Supplementary file 3 — Supplementary material 3 (PPTX 600 kb) [file 12350_2017_813_MOESM3_ESM.pptx]

## Slide 1
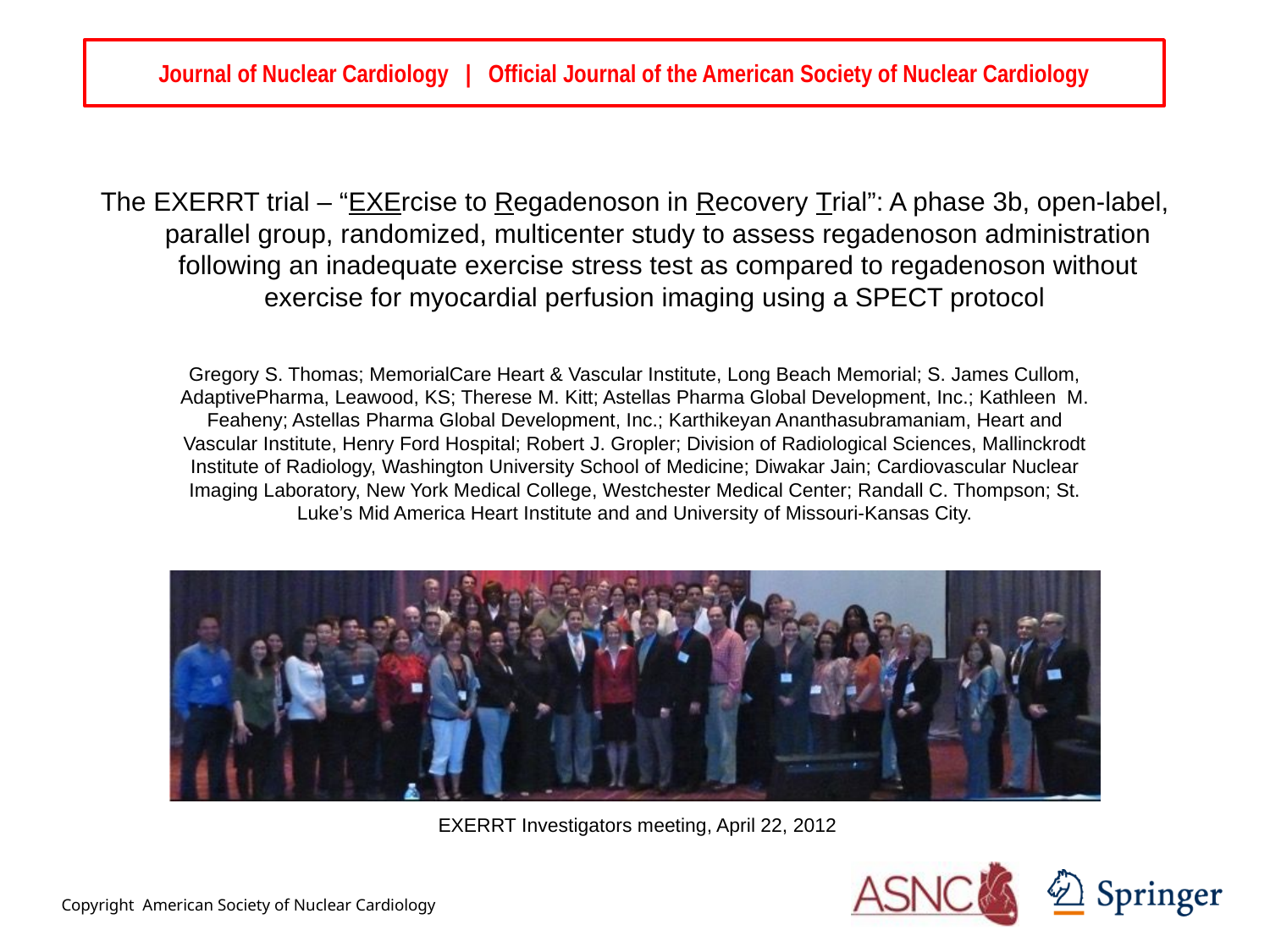

Journal of Nuclear Cardiology | Official Journal of the American Society of Nuclear Cardiology
# The EXERRT trial – “EXErcise to Regadenoson in Recovery Trial”: A phase 3b, open-label, parallel group, randomized, multicenter study to assess regadenoson administration following an inadequate exercise stress test as compared to regadenoson without exercise for myocardial perfusion imaging using a SPECT protocol
Gregory S. Thomas; MemorialCare Heart & Vascular Institute, Long Beach Memorial; S. James Cullom, AdaptivePharma, Leawood, KS; Therese M. Kitt; Astellas Pharma Global Development, Inc.; Kathleen M. Feaheny; Astellas Pharma Global Development, Inc.; Karthikeyan Ananthasubramaniam, Heart and Vascular Institute, Henry Ford Hospital; Robert J. Gropler; Division of Radiological Sciences, Mallinckrodt Institute of Radiology, Washington University School of Medicine; Diwakar Jain; Cardiovascular Nuclear Imaging Laboratory, New York Medical College, Westchester Medical Center; Randall C. Thompson; St. Luke’s Mid America Heart Institute and and University of Missouri-Kansas City.
EXERRT Investigators meeting, April 22, 2012
Copyright American Society of Nuclear Cardiology

## Slide 2
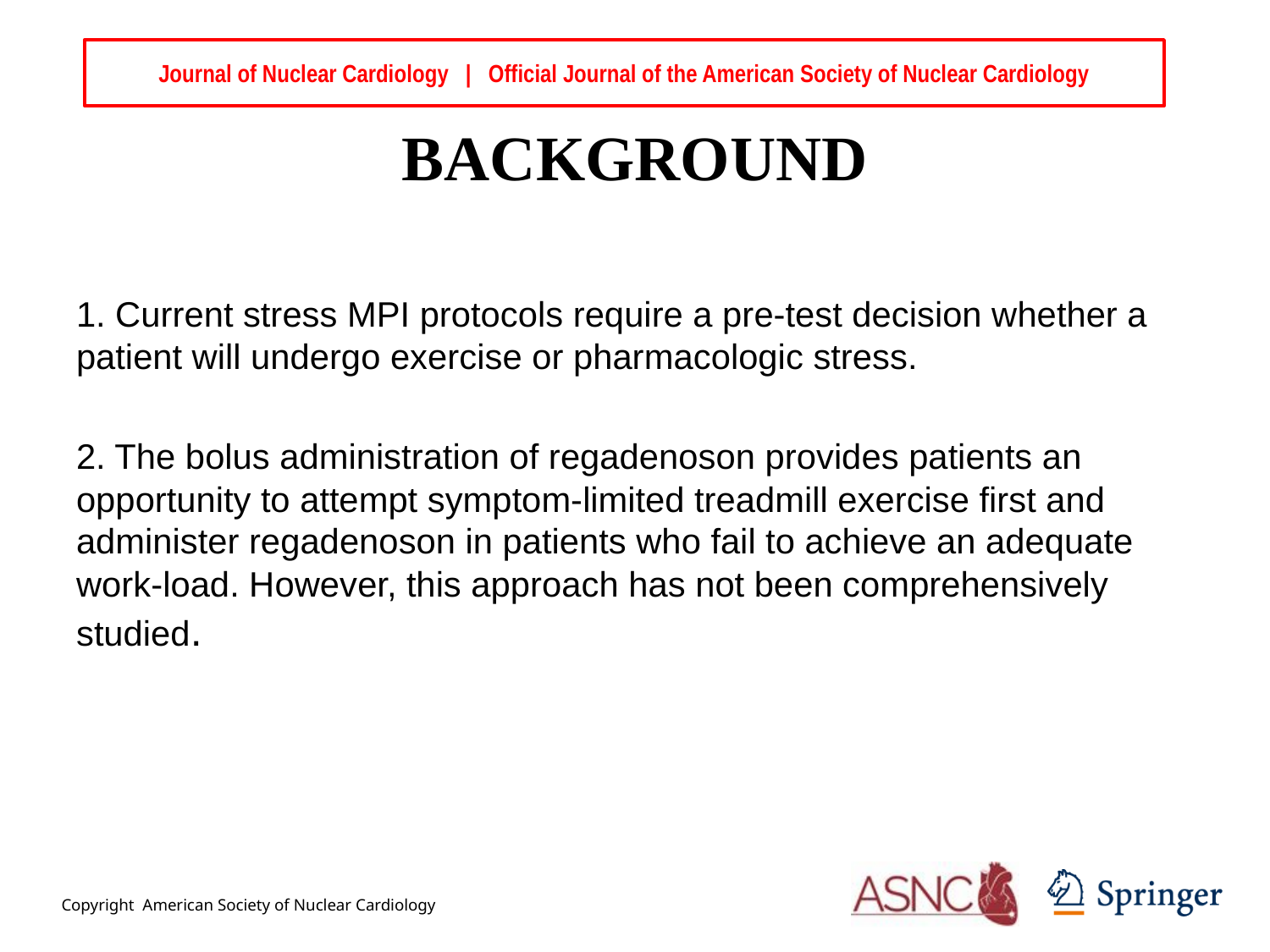

Journal of Nuclear Cardiology | Official Journal of the American Society of Nuclear Cardiology
# BACKGROUND
1. Current stress MPI protocols require a pre-test decision whether a patient will undergo exercise or pharmacologic stress.
2. The bolus administration of regadenoson provides patients an opportunity to attempt symptom-limited treadmill exercise first and administer regadenoson in patients who fail to achieve an adequate work-load. However, this approach has not been comprehensively studied.
Copyright American Society of Nuclear Cardiology

## Slide 3
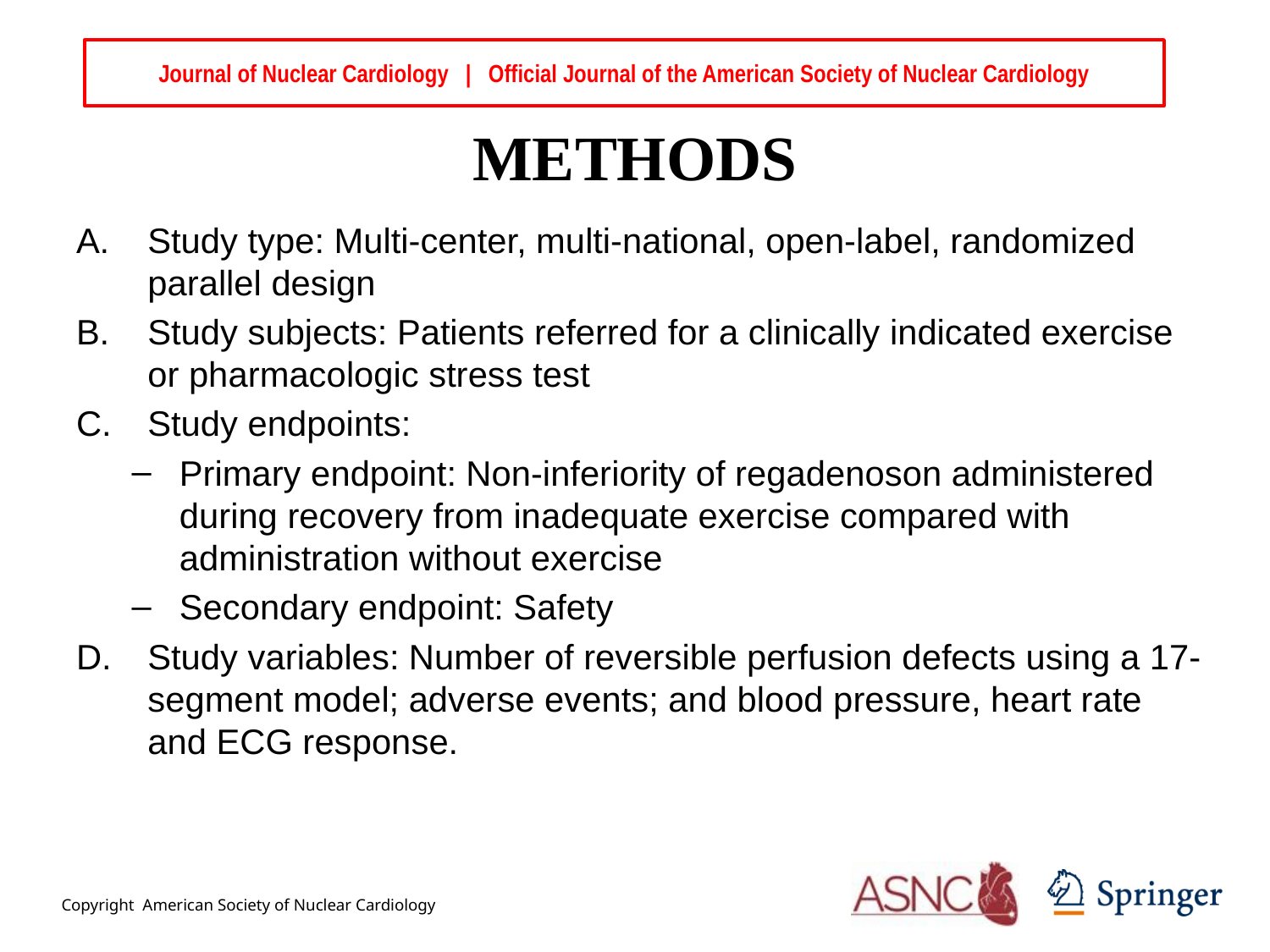

Journal of Nuclear Cardiology | Official Journal of the American Society of Nuclear Cardiology
# METHODS
Study type: Multi-center, multi-national, open-label, randomized parallel design
Study subjects: Patients referred for a clinically indicated exercise or pharmacologic stress test
Study endpoints:
Primary endpoint: Non-inferiority of regadenoson administered during recovery from inadequate exercise compared with administration without exercise
Secondary endpoint: Safety
Study variables: Number of reversible perfusion defects using a 17-segment model; adverse events; and blood pressure, heart rate and ECG response.
Copyright American Society of Nuclear Cardiology

## Slide 4
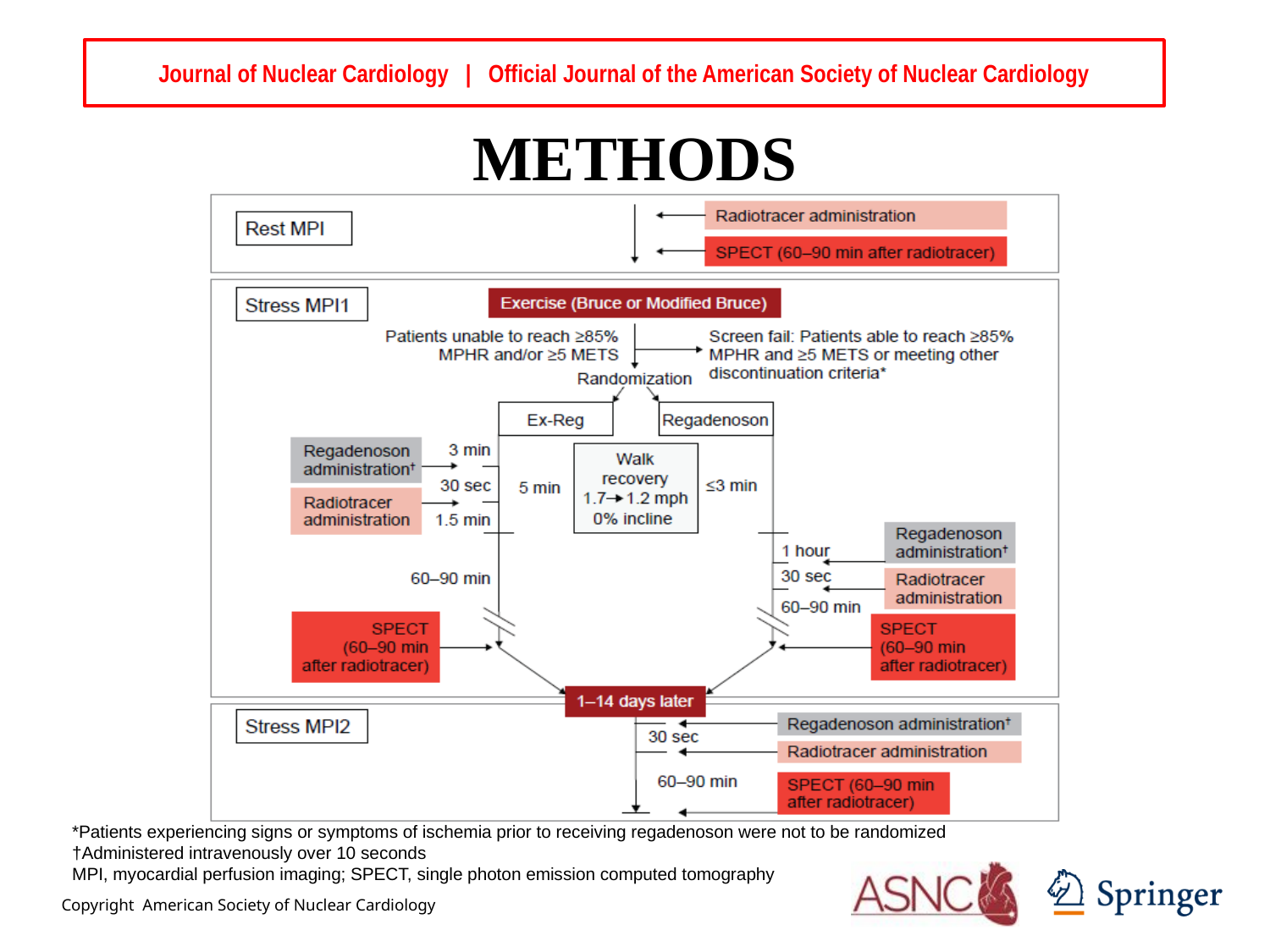

Journal of Nuclear Cardiology | Official Journal of the American Society of Nuclear Cardiology
# METHODS
*Patients experiencing signs or symptoms of ischemia prior to receiving regadenoson were not to be randomized
†Administered intravenously over 10 seconds
MPI, myocardial perfusion imaging; SPECT, single photon emission computed tomography
Copyright American Society of Nuclear Cardiology

## Slide 5
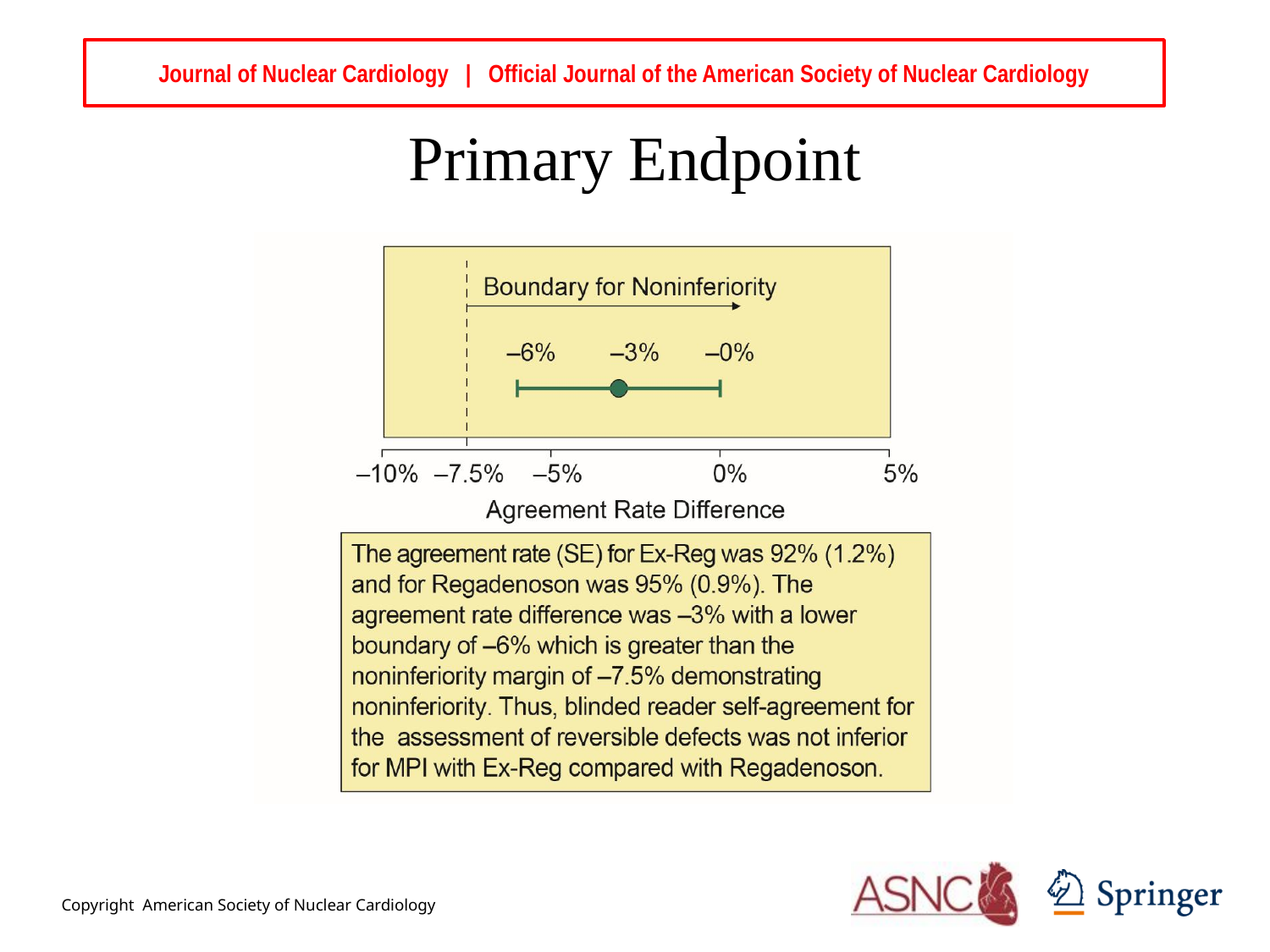

Journal of Nuclear Cardiology | Official Journal of the American Society of Nuclear Cardiology
# Primary Endpoint
Copyright American Society of Nuclear Cardiology

## Slide 6
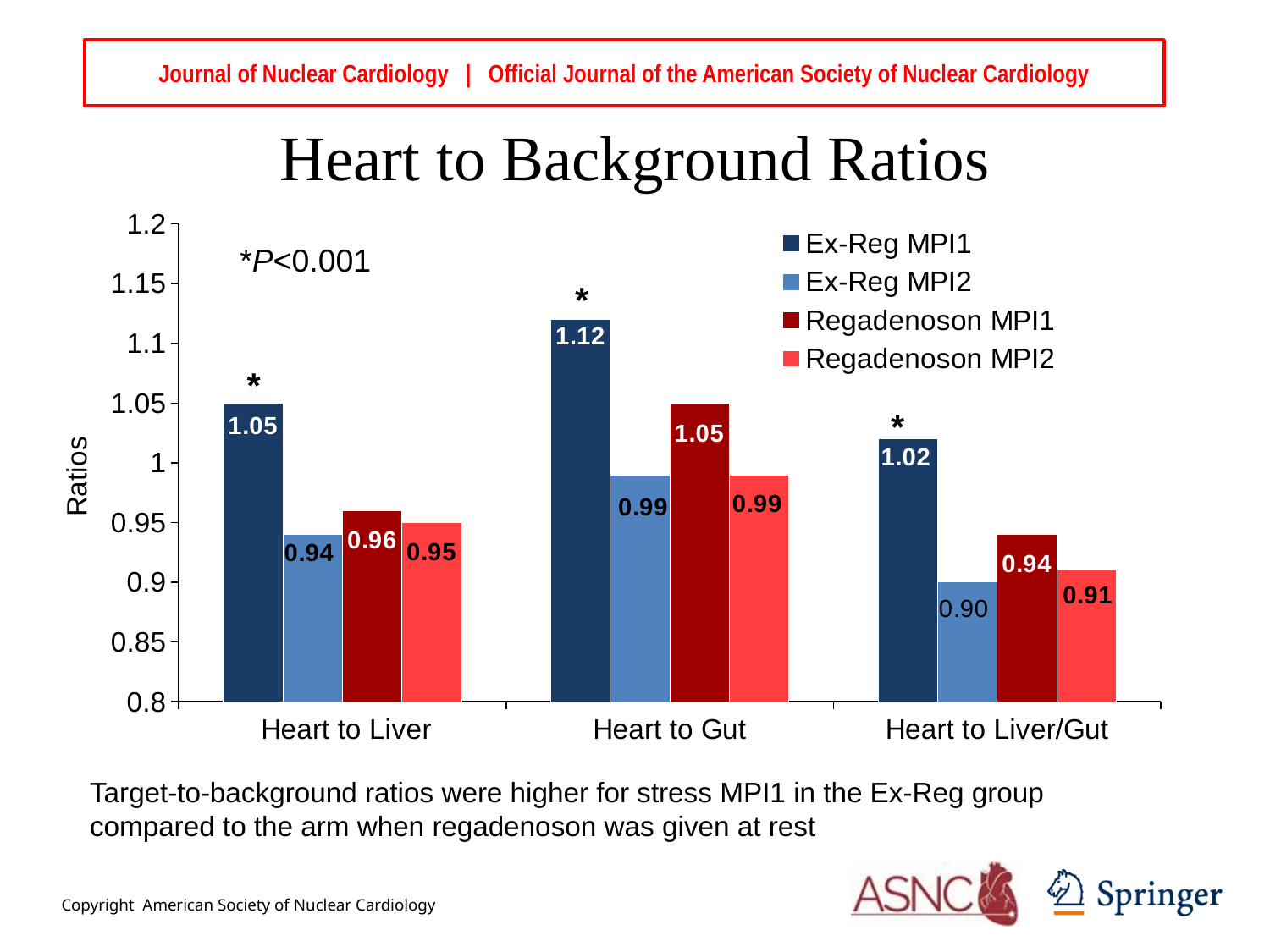

Journal of Nuclear Cardiology | Official Journal of the American Society of Nuclear Cardiology
# Heart to Background Ratios
### Chart
| Category | Ex-Reg MPI1 | Ex-Reg MPI2 | Regadenoson MPI1 | Regadenoson MPI2 |
|---|---|---|---|---|
| Heart to Liver | 1.05 | 0.94 | 0.96 | 0.95 |
| Heart to Gut | 1.12 | 0.99 | 1.05 | 0.99 |
| Heart to Liver/Gut | 1.02 | 0.9 | 0.94 | 0.91 |*P<0.001
Target-to-background ratios were higher for stress MPI1 in the Ex-Reg group compared to the arm when regadenoson was given at rest
Copyright American Society of Nuclear Cardiology

## Slide 7
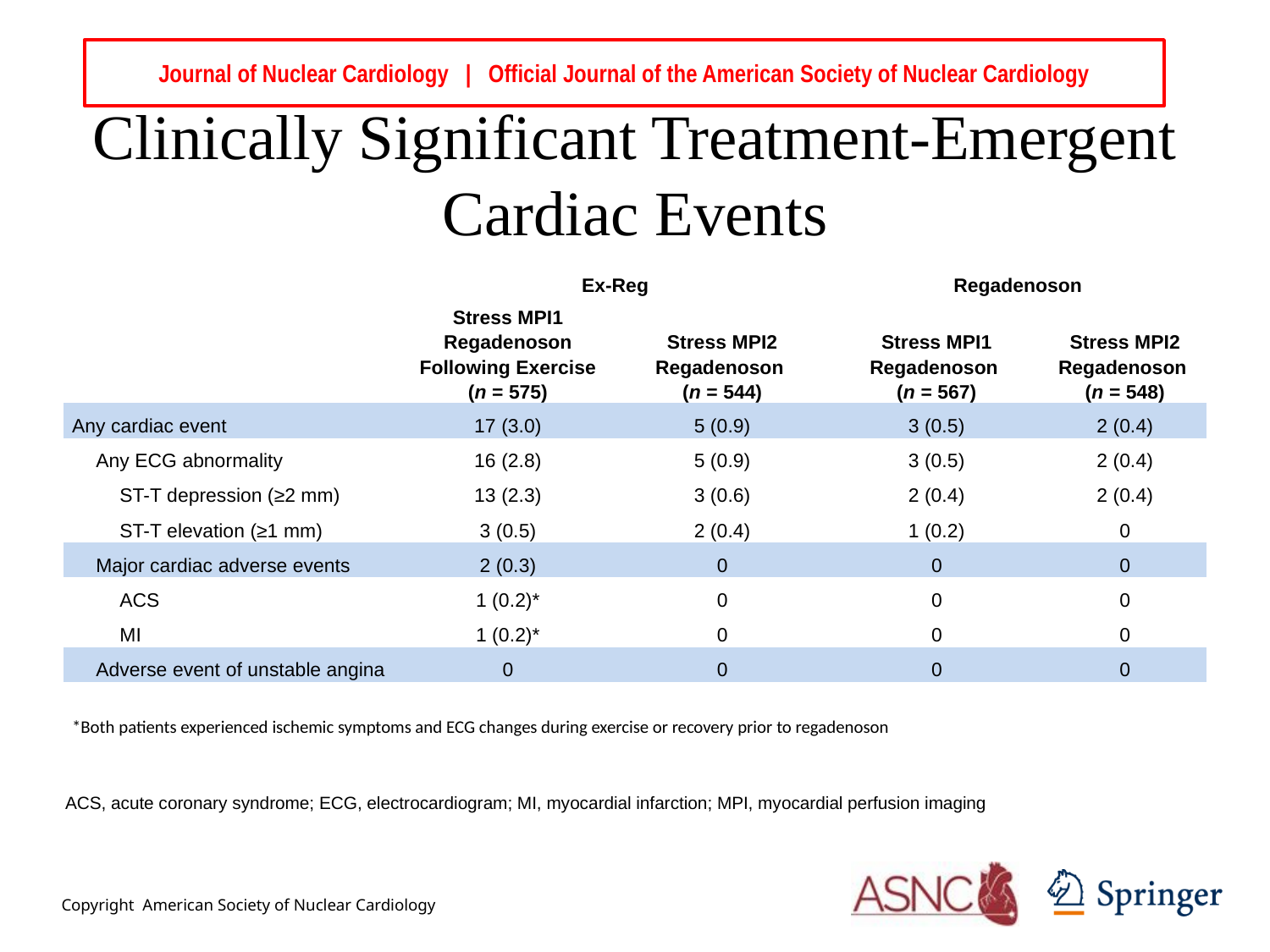

Journal of Nuclear Cardiology | Official Journal of the American Society of Nuclear Cardiology
# Clinically Significant Treatment-Emergent Cardiac Events
| | Ex-Reg | | Regadenoson | |
| --- | --- | --- | --- | --- |
| | Stress MPI1 Regadenoson Following Exercise (n = 575) | Stress MPI2 Regadenoson (n = 544) | Stress MPI1 Regadenoson (n = 567) | Stress MPI2 Regadenoson (n = 548) |
| Any cardiac event | 17 (3.0) | 5 (0.9) | 3 (0.5) | 2 (0.4) |
| Any ECG abnormality | 16 (2.8) | 5 (0.9) | 3 (0.5) | 2 (0.4) |
| ST-T depression (≥2 mm) | 13 (2.3) | 3 (0.6) | 2 (0.4) | 2 (0.4) |
| ST-T elevation (≥1 mm) | 3 (0.5) | 2 (0.4) | 1 (0.2) | 0 |
| Major cardiac adverse events | 2 (0.3) | 0 | 0 | 0 |
| ACS | 1 (0.2)\* | 0 | 0 | 0 |
| MI | 1 (0.2)\* | 0 | 0 | 0 |
| Adverse event of unstable angina | 0 | 0 | 0 | 0 |
| \*Both patients experienced ischemic symptoms and ECG changes during exercise or recovery prior to regadenoson | | | | |
ACS, acute coronary syndrome; ECG, electrocardiogram; MI, myocardial infarction; MPI, myocardial perfusion imaging
Copyright American Society of Nuclear Cardiology

## Slide 8
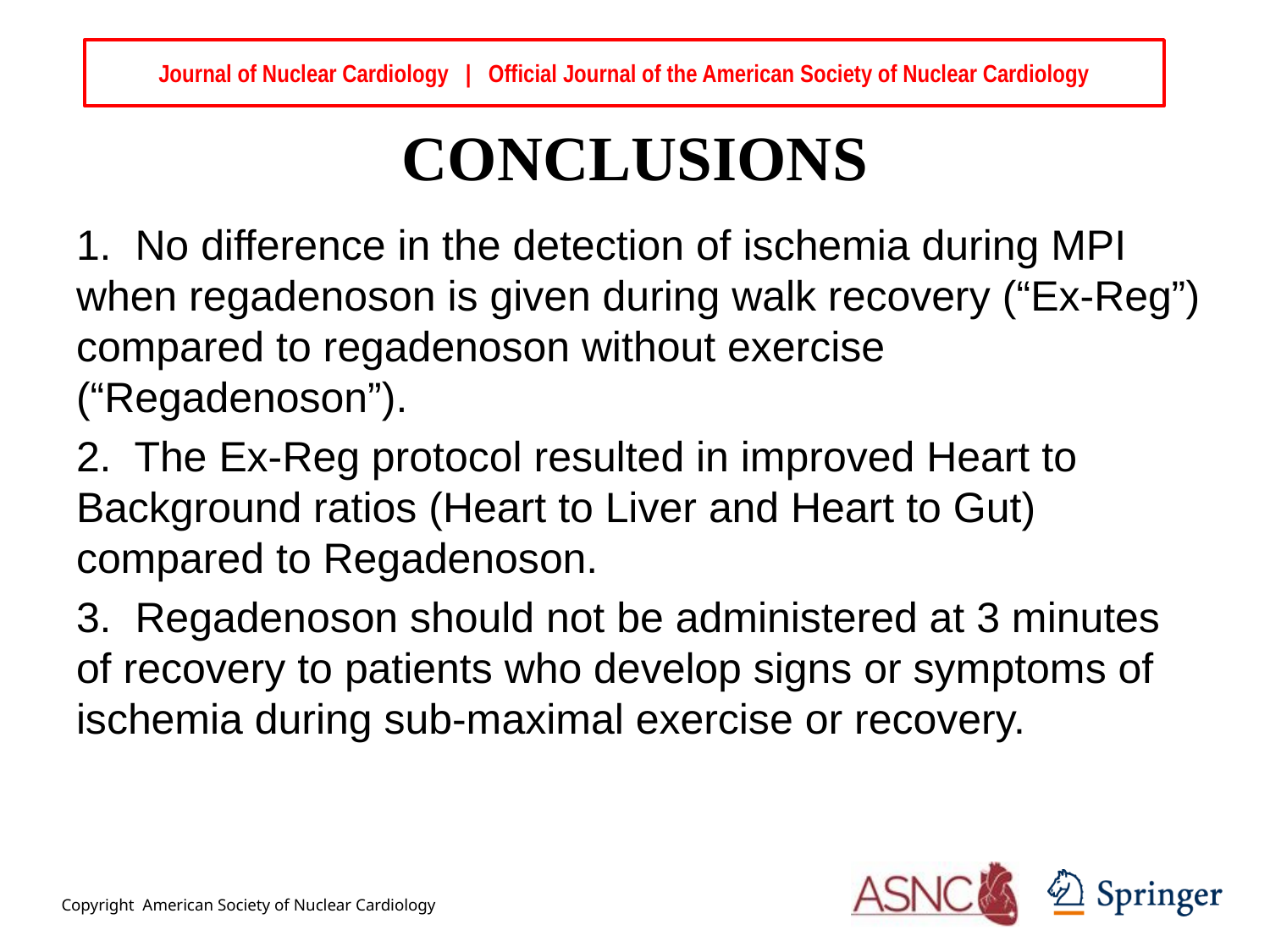

Journal of Nuclear Cardiology | Official Journal of the American Society of Nuclear Cardiology
# CONCLUSIONS
1. No difference in the detection of ischemia during MPI when regadenoson is given during walk recovery (“Ex-Reg”) compared to regadenoson without exercise (“Regadenoson”).
2. The Ex-Reg protocol resulted in improved Heart to Background ratios (Heart to Liver and Heart to Gut) compared to Regadenoson.
3. Regadenoson should not be administered at 3 minutes of recovery to patients who develop signs or symptoms of ischemia during sub-maximal exercise or recovery.
Copyright American Society of Nuclear Cardiology
